# The role of CsrA in controls the extracellular electron transfer and biofilm production in *Geobacter sulfurreducens*

**DOI:** 10.3389/fmicb.2025.1534446

**Published:** 2025-03-11

**Authors:** Alberto Hernández-Eligio, Leticia Vega-Alvarado, Xinying Liu, Jessica Cholula-Calixto, Guillermo Huerta-Miranda, Katy Juárez

**Affiliations:** ^1^Departamento de Microbiología Molecular, Instituto de Biotecnología, Universidad Nacional Autónoma de México, Cuernavaca, Morelos, México; ^2^Investigador por México, Consejo Nacional de Humanidades Ciencia y Tecnologías, Ciudad de México, México; ^3^Instituto de Ciencias Aplicadas y Tecnología, Universidad Nacional Autónoma de México, Ciudad Universitaria, Ciudad de México, México; ^4^Beijing Key Laboratory for Source Control Technology of Water Pollution, Engineering Research Center for Water Pollution Source Control and Eco-remediation, College of Environmental Science and Engineering, Beijing Forestry University, Beijing, China

**Keywords:** CsrA post-transcriptional regulator, RNA-seq, biofilm, microbial fuel cell, current production

## Abstract

CsrA is a post-transcriptional regulator that controls biofilm formation, virulence, carbon metabolism, and motility, among other phenotypes in bacteria. CsrA has been extensively studied in γ-proteobacteria and firmicutes, However the cellular processes controlled for regulation in δ-proteobacteria remain unknown. In this work, we constructed and characterized the Δ*csrA* mutant strain in *Geobacter sulfurreducens* to determine the involvement of the CsrA protein in the regulation of biofilm and extracellular electron transfer. The Δ*csrA* mutant strain shows higher rates of insoluble Fe(III) reduction than the wild type using acetate as electron donor and the growth with fumarate and soluble (Fe(III)) was similar to wild type. Biofilm quantification and characterization by confocal laser scanning microscopy, showed that the Δ*csrA* mutant produces up to twice as much biofilm as the wild type strain and more than 95% viable cells. Transcriptome analysis by RNA-seq showed that in Δ*csrA* biofilms developed on an inert support, differentially expressed 244 genes (103 upregulated and 141 downregulated), including those related to extracellular electron transfer, exopolysaccharide synthesis, c-di-GMP synthesis and degradation. To validate the transcriptome data, RT-qPCR confirmed the differential expression of several selected genes in the Δ*csrA* strain. Also, current production in microbial fuel cells was performed and the Δ*csrA* strain produced 45–50% more current than the wild type. To identify the genes that changed expression in the Δ*csrA* strain in the graphite electrodes in an MFC, a transcriptome analysis was performed 181 genes changed their expression in the Δ*csrA* biofilms, of which 113 genes were differentially expressed only in MFC and 68 genes changed their expression as well as the transcriptome of biofilms grown on glass. *In silico* analysis of the 5′-UTR regions revealed that 76 genes that changed expression in the RNA-seq analysis have a consensus sequence for CsrA binding. To our knowledge this is the first report describing the involvement of CsrA in the regulation of extracellular electron transfer and biofilm in a member of the δ-proteobacteria.

## 1 Introduction

Bacteria have developed strategies to contend and adapt to diverse environmental challenges. As part of this response to various stimuli, bacteria change the expression of genes required for a given state, where transcriptional and post-transcriptional regulators are essential for coordinating gene expression patterns. Carbon storage regulator A (CsrA) is one of the most studied post-transcriptional regulators in bacteria. The global regulator CsrA controls a wide variety of cellular processes, including carbon metabolism (Morin et al., 2016; Revelles et al., 2013), biofilm formation (Wang et al., 2005; Silva-Rohwer et al., 2023), virulence (Vakulskas et al., 2015), motility (Wei et al., 2001; Oshiro et al., 2020), quorum sensing (Lenz et al., 2005), and stress response (Potts et al., 2019), among others. The regulatory mechanism of CsrA is based on its binding near or overlapping the Shine-Dalgarno ribosome binding site of the target messenger RNA (mRNA), promoting changes in structure, translation, stability, and translational elongation (Potts et al., 2019). CsrA binds to site-specific sequences in the mRNA, where the conserved GGA motif is present in a stem-and-loop structure, and is critical for RNA-protein interaction (Renda et al., 2020). Several studies in *Escherichia coli, Salmonella typhi*, and *Pseudomonas aeruginosa* show that CsrA is primarily a translational repressor, but there are examples where CsrA binds to the 5′-Untranslated Region (5′-UTR) of the *flhDC* mRNA to stabilize it, inhibiting its degradation by RNase E and thus promoting the expression of the master regulator of genes involved in flagellum biosynthesis (Vakulskas et al., 2015; Yakhnin et al., 2013). In γ-proteobacteria, CsrA is regulated by small noncoding RNAs of the CsrB/CsrC family. The noncoding RNA molecule employs multiple high-affinity binding sites to sequester an mRNA-binding regulatory protein away from its target mRNAs. On the other hand, in the firmicute *Bacillus subtilis* and the ε-proteobacterium *Campylobacter jejuni*, CsrA inhibits translation of the gene encoding the flagellin protein, but unlike in γ-proteobacteria, CsrA is regulated by interaction with the FliW protein (Bogacz et al., 2021; Oshiro et al., 2020). Although CsrA has been extensively studied in γ-proteobacteria and the CsrA protein is highly conserved among bacterial species, its function in δ-proteobacteria is unknown.

*Geobacter sulfurreducens* is a δ-proteobacterium living in the subsurface environment. This bacterium can couple the oxidation of organic compounds with the reduction of Fe(III) and Mn(IV) present in soil and sediments (Lovley et al., 2011). *G. sulfurreducens* can transfer its electrons beyond the cell to extracellular acceptors such as metals, electrodes in microbial fuel cells, and other microorganisms through a process called extracellular electron transfer (Bond and Lovley, 2003; Aklujkar et al., 2013; Rotaru et al., 2014). For extracellular electron transfer, *G. sulfurreducens* uses a set of *c*-type cytochrome proteins located in the inner membrane, periplasm, and outer membrane, in addition to cytochrome nanowires and extracellular conductive pili (Reguera et al., 2005; Ueki, 2021; Liu et al., 2021; Schwarz et al., 2024). *G. sulfurreducens* is a model for the study of extracellular electron transfer mechanisms, its genome has been sequenced and genetic tools for its manipulation have been developed (Methé et al., 2003; Coppi et al., 2001).

*G. sulfurreducens* is one of the most efficient bacteria to produce high power densities in microbial fuel cells (Nevin et al., 2008; Malvankar et al., 2012). The potential to produce high current densities is attributed to the fact that *G. sulfurreducens* can develop highly conductive biofilms in which extracellular long-range electron transfer is an indispensable process and is mainly driven by a network of pilin nanowires (Nevin et al., 2009; Malvankar et al., 2012; Wang et al., 2024). Other elements involved in the formation of conductive biofilms are *c*-type cytochromes, lipids, flagella, and exopolysaccharides (Nevin et al., 2009; Steidl et al., 2016; Liu et al., 2019). Mutations in genes encoding proteins involved in exopolysaccharide synthesis (GSU1501), cytochromes, and pili severely affect current production (Rollefson et al., 2011). Conversely, overexpression of the GSU1501 gene in *G. sulfurreducens* increases exopolysaccharide production, biofilm formation and current production (Zhuang et al., 2020). Despite the importance of conductive biofilm formation on electrodes in microbial fuel cells for the current generation, little is known about the regulatory mechanisms and protein regulators that control the genes involved in biofilm formation in *G. sulfurreducens*.

In this work, we investigated the role of CsrA in *G. sulfurreducens* and its involvement in regulating electroactive biofilm formation and extracellular electron transfer. To address the above questions, we generated the *csrA* mutant strain and characterized its biofilms using confocal laser scanning microscopy (CLSM). RNA-seq of biofilms of the Δ*csrA* strain grown on non-conductive supports reveals that 244 genes change their expression. We show that in microbial fuel cells, Δ*csrA* produces 40% more energy than the wild-type strain, and RNA-seq analysis of biofilms grown on MFC shows that 181 genes change their expression. Among the genes that changed their expression were those encoding *c*-type cytochromes, enzymes involved in exopolysaccharide synthesis, transcriptional regulators, hydrogenases, and efflux pumps. Our results demonstrate that in *G. sulfurreducens* CsrA is a global regulator that controls extracellular electron transfer and biosynthesis of conductive biofilms.

## 2 Materials and methods

### 2.1 Culture conditions, bacteria, and plasmids

The *G. sulfurreducens* strains (DL1 and Δ*csrA* mutant) (Supplementary Table 1) were routinely cultured anaerobically in either NBAF medium (20 mM of acetate and fumarate 40 mM, as donor and acceptor electron, respectively) or acetate-Fe(III) citrate medium (20 and 50 mM, respectively), as previously described (Coppi et al., 2001). Anoxic sterile antibiotic (300 μg/mL kanamycin) was added to acetate-fumarate plates during mutant strain selection. For growth curves and Fe(III) reduction assays *G. sulfurreducens* were incubated at 30°C. For biofilm grown on glass (inert support) or FTO supports, the *G. sulfurreducens* strains were incubated in NBAF medium without shaking at 25°C. *E. coli* strains DH5α, and S17-1 (Supplementary Table 1), were used for DNA manipulations, and for conjugation experiments, respectively.

### 2.2 Construction of Δ*csrA* mutant

To construct the *G. sulfurreducens* Δ*csrA* mutant strain we used the markerless deletion method (Chan et al., 2015). The flanking regions (794-bp upstream and 706-bp downstream) of *csrA* were amplified with the csrAFw1Bam/csrARv2quim and csrAFw3quim/csrARr4Eco oligonucleotides pairs (Supplementary Table 1) using genomic DNA as the template coupled with the Phusion High-Fidelity DNA Polymerase (Thermo). The flanking regions of *csrA* were joined in the second round of PCR, digested with *Bam*HI and *Eco*RI, and ligated into the same sites in the pK18mobsacB plasmid to generate pK18mobsacB-csrAdel.

The pK18mobsacB-csrAdel plasmid was transformed into the *E. coli* conjugative donor strain S17-1 to conjugate into the *G. sulfurreducens*. One milliliter of *G. sulfurreducens* (OD600 0.3) acetate-fumarate culture was pelleted on top of 1 ml of S17-1 culture carrying the pK18mobsacB-csrAdel plasmid, mixed on 0.22-μm-pore-size filters resting on acetate-fumarate agar plates in an anaerobic chamber, and incubated for 4 h, after which the mixture was streaked onto Km-containing acetate-fumarate plates. This procedure was followed to select *G. sulfurreducens* cultures with pK18mobsacB-csrAdel integrated into either flanking region of the target gene, as the plasmid cannot replicate in *G. sulfurreducens*. A gene deletion was selected on acetate-fumarate plates containing 10% sucrose and confirmed using PCR with primers flanking the deletion site (Supplementary Figure 1).

### 2.3 Complementation of Δ*csrA* mutant

To complement the Δ*csrA* mutant strain the pRG5.1-RRflg-*csrA* plasmid was constructed. This plasmid contains the *csrA* gene fused to the regulatory region of *flgJ* gene (RRflg). First, a 218- and 253-bp DNA fragments containing the RRflg and *csrA* gene, respectively, were amplified separately from chromosomal DNA using Phusion High-Fidelity DNA Polymerase coupled with RRflgEcoRIfw/RRflgNdeIrev and csrAFwNdeI/csrAHindIIIrev primers. The 218-bp RRflg was cloned into the pJET1.2 plasmid (Thermo) to generate the pJET-RRflg plasmid. The *csrA* gene was digested with *Nde*I and *Hind*III, and cloned into a similarly digested pJET-RRflg, resulting in pJET-RRflg-*csrA*. Then, *csrA* fused to RRflg was released from pJET-RRflg-*csrA* by digestion with *Eco*RI and *Hind*III, and cloned into similarly digested pRG5.1 giving rise to pRG5.1-RRflg-*csrA*. The pRG5.1-RRflg-*csrA* plasmid was sequenced to confirm the presence of an intact RRflg-*csrA* fusion and transformed into DL1 and Δ*csrA* strains.

### 2.4 RNA extraction

*G. sulfurreducens* cells recovered from biofilm both grown in glass and graphite electrode-respiring conditions were used for RNA-seq and RT-qPCR analyses. Briefly, for the cells from biofilm grown in glass, the culture was incubated in NBAF medium for 48 h at 25°C. Afterwards, the planktonic cells were separated from the biofilms formed at the bottom of the culture bottle (glass), and this was recovered with 1 mL to fresh NBAF and 100 μL RNAlater (Thermo). For the biofilms grown on a graphite electrode, the biofilm from anode assembled in an H-type MCF with FWAF medium was washed with PBS buffer and collected with 1 mL of FWAF and 100 μL of RNA*later* (Thermo). In both cases, the cells were stored at −70°C until use. For each biological sample, total RNA samples were extracted using the RNeasy mini kit (Qiagen), then they were examined with an Agilent 2100 Bioanalyzer and quantified using NanoDrop 200c (Thermo). The DNA contaminant was eliminated using DNase I (RNAse free) (Thermo).

### 2.5 RNA-seq analysis

RNA-Seq was performed using RNA samples extracted from *G. sulfurreducens* biofilms (DL1 and Δ*csrA* strains) from glass and anode electrode by duplicate, using independent samples. Illumina sequencing was performed at UUSMB (Unidad Universitaria de Secuenciación Masiva y Bioinformatica, UNAM, México). Briefly, ribosomal RNA was depleted using the Ribominus kit (Thermo), and the mRNA-enriched RNA was chemically fragmented to 150–200 bp. Based on these cleaved RNA fragments, cDNA libraries were synthesized using TruSeq Stranded mRNA kit (Illumina), after which they were purified using the Zymoclean Gel DNA Recovery Kit (Zymo Research). Libraries were sequenced on an Illumina NextSeq 500 sequencer. Differential expression analyses were performed through IDEAmex website (http://www.uusmb.unam.mx/ideamex/) (Jiménez-Jacinto et al., 2019) using four methods: edgeR (Robinson and Oshlack, 2010), DESeq2 (Anders and Huber, 2010), NOISeq (Tarazona et al., 2011), and limma (Ritchie et al., 2015). To identify differentially expressed genes, we selected those whose *p*-value were < 0.05 and fold change > 1.5, for each method. We considered as the best candidates, only genes that appeared differentially expressed in the four methods. The functional annotation of differentially expressed genes was obtained from Kyoto Encyclopedia of Genes and Genomes (KEGG) (Kanehisa and Goto, 2000), using our own R's scripts. RNA-seq transcriptome data were deposited in the NCBI Gene Expression Omnibus database under accession number GSE282747. Principal component analysis identified clustering of Δ*csrA* mutant strain with respect to DL1 strain both glass (Supplementary Figure 2A) and anode electrode (Supplementary Figure 2B).

### 2.6 Reverse transcription and quantitative PCR (RT-qPCR)

To validate the quality of RNA-seq data, 22 differentially expressed genes (nine upregulated and 13 downregulated) from biofilms grown in glass were selected for RT-qPCR analysis. mRNA was extracted as described in the section “RNA extraction”. cDNA synthesis was performed using 1 μg total RNA (DNA free), 20 pmol reverse primers specific for each gene evaluated, and the RevertAid H Minus First Strand cDNA Synthesis Kit (Thermo). Previously, the primers were evaluated for specific amplification by point-end PCR with genomic DNA (Supplementary Figure 3). Subsequently, qPCR was performed using a Maxima SYBR Green/ROX qPCR Master Mix (Thermo) with the Rotor-Gene Q (Qiagen). We used the next program: 95°C for 10 min, followed by 40 cycles of 95°C for 15 s, 60°C for 60 s. Gene-specific oligonucleotides used for RT-qPCR are shown in Supplementary Table 1, *recA* was used as an internal gene standard for PCR amplification. Normalized fold changes of the relative expression ratio were quantified using the 2^−ΔΔCT^ method (Livak and Schmittgen, 2001). All experiments were performed in triplicate, using independent samples, and their average values were calculated.

### 2.7 Analysis of biofilm and structure by CLSM

The biofilm structure and the ratio of live cells to dead cells were determined by confocal laser scanning microscopy (CLSM), as a previous report (Hernández-Eligio et al., 2022; Jaramillo-Rodríguez et al., 2023; Rodríguez-Torres et al., 2024). Fluorine-doped tin oxide (FTO) electrodes were used as supports for biofilm formation inside hermetically sealed test tubes in anaerobic conditions with NBAF medium as described in Culture conditions section. After removing the supports from the culture medium, the planktonic cells were removed from the biofilm with a mixture of 0.002 M cysteine and 0.9% saline isotonic solution (anoxic and sterile). Afterward, a mixture of dyes from the LIVE/DEAD^®^ BacLight Bacterial Viability kit (0.00334 mM SYTO9 and 0.02 M propidium iodide) dissolved in 0.9% saline isotonic solution and 0.1 M cysteine was added to the samples. The samples were dyed for 10 min, during which they were protected from any extraneous light sources. The dye was then washed with 0.002 M cysteine and 0.9% saline solution. Finally, images were captured with an Olympus FV1000 microscope at excitation wavelengths of 488 nm (green channel) and 559 nm (red channel). Imaging was performed using an immersion objective (LUMFLN 60 X 1.1 W). Fluorescence was obtained with a spectral detector at a 500–545 nm range (SDM560) for the green channel and a 570–670 nm range (Mirror) for the red channel. Images were acquired through the *Z*-axis of the biofilm at regular thickness intervals. Image analysis was performed using the Comstat2 (version 2.1) and Fiji (version 2.9.0) software (Heydorn et al., 2000; Schindelin et al., 2012).

### 2.8 Current production in microbial fuel cell

The current production of Δ*csrA* and wild type strains were compared in a two-chambered H-cell system as previously reported (Nevin et al., 2009). Graphite electrodes as the electron donor and graphite stick anodes (65 cm^2^) poised with a potentiostat 300 mV vs. Ag/AgCl as the electron acceptor. The cells were grown with acetate (10 mM)-fumarate (40 mM) medium, and once current production was initiated, the anode chamber received a steady input of fresh medium containing acetate (10 mM) and no fumarate. Current measurements were collected from potentiostat and data was analyzed with Chart 5 software. A schematic diagram of the MFC as shown in Supplementary Figure 4.

## 3 Results and discussion

### 3.1 Genetic organization of the *csrA* locus in *G. sulfurreducens*

**Figure 1:**
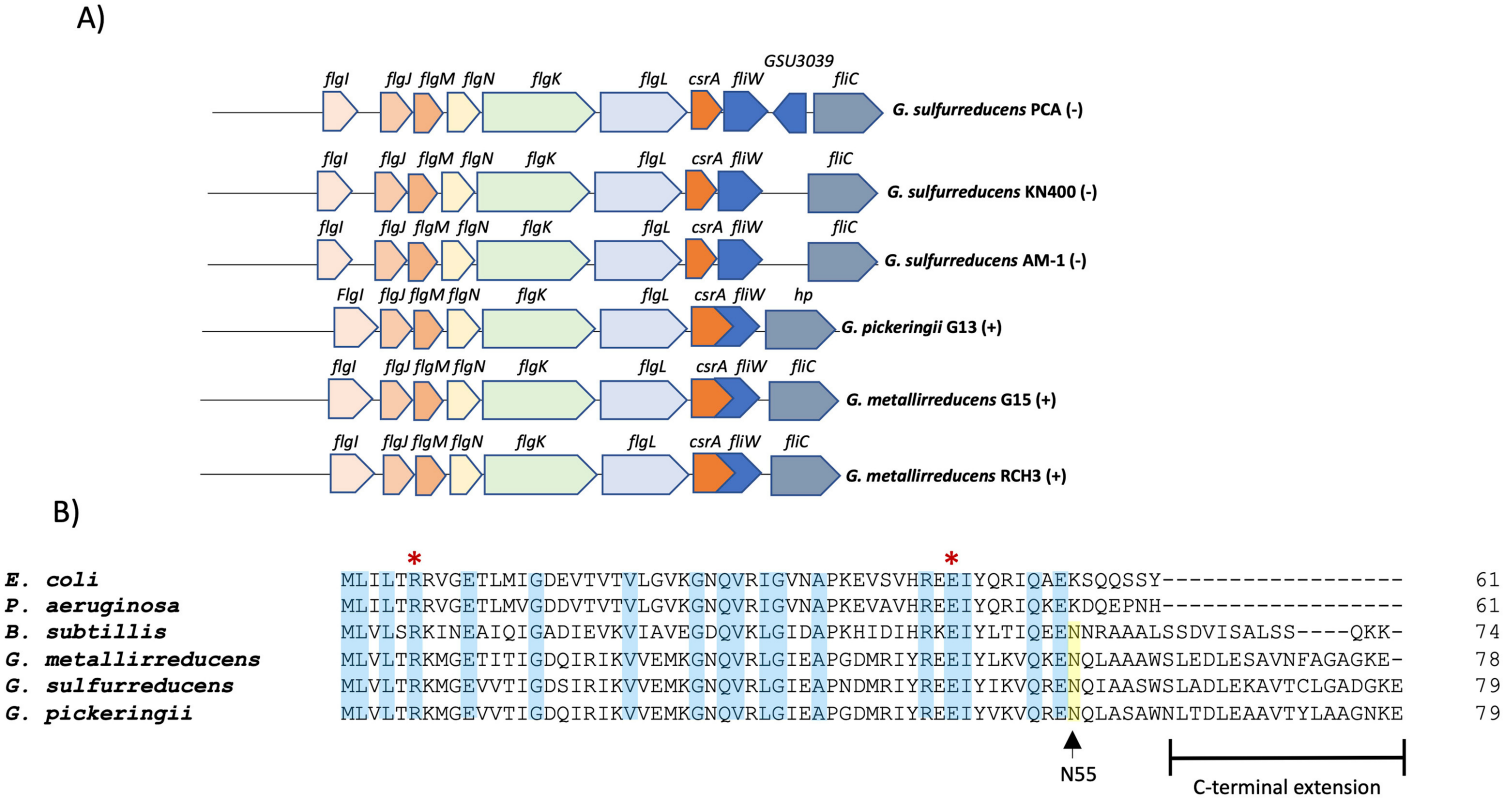
CsrA in *G. sulfurreducens*. **(A)** Organization and conservation of the csrA locus in different Geobacter species. The symbol “(+)” indicates that it is encoded in the positive strand and “(–)” indicates that it is encoded in the negative strand. **(B)** Primary sequence alignment of CsrA proteins from selected species: *Escherichia coli* (*E. coli*), *Pseudomonas aeruginosa* (*P. aeruginosa*), *Bacillus subtilis* (*B. subtilis*), *Geobacter sulfurreducens* (*G. sulfurreducens*), *Geobacter metallireducens* (*G. metallireducens*), *Geobacter pickeringii* (*G. pickeringii*). The blue box indicates positions with a fully conserved residue; the yellow box indicates the conserved N55 asparagine; the red asterisk indicates the conserved arginine (R) and glutamic acid (E) which are essential for RNA-binding activity in CsrA proteins.

The *csrA* gene is conserved among different *Geobacter* species (*G. sulfurreducens, G. metallireducens*, and *G. pickeringii*). In these bacteria, the *csrA* gene is part of a putative operon (*flgJMNKL-csrA-fliW*) with six genes that encode flagellar proteins (Figure 1A). The *csrA* locus is encoded on the negative strand in *G. sulfurreducens* strains (PCA, AM-1, KN400), and on the positive strand in *G. metallireducens* and *G. pickeringii*. In the latter, *csrA* overlaps with the *fliW* gene. Among *G. sulfurreducens* species (PCA, KN400, AM-1), CsrA shows 100% identity, while compared to *G. pickeringii* and *G. metallireducens* (GS-15), CsrA shows 83.54 and 82.25% identity, respectively. In contrast, *G. sulfurreducens* CsrA shares 45.9% identity with *E. coli* CsrA protein and 50% identity with *B. subtilis* CsrA. The residues arginine (R6) and glutamic acid (E46) involved in the regulatory activity of *E. coli* CsrA are conserved in *G. sulfurreducens* CsrA. In addition, the protein has a C-terminal extension and a conserved asparagine (N55) residue required for FliW antagonism in *B. subtilis* and *C. jejuni* (Mukherjee et al., 2016; Bogacz et al., 2021) (Figure 1B).

**Figure 2:**
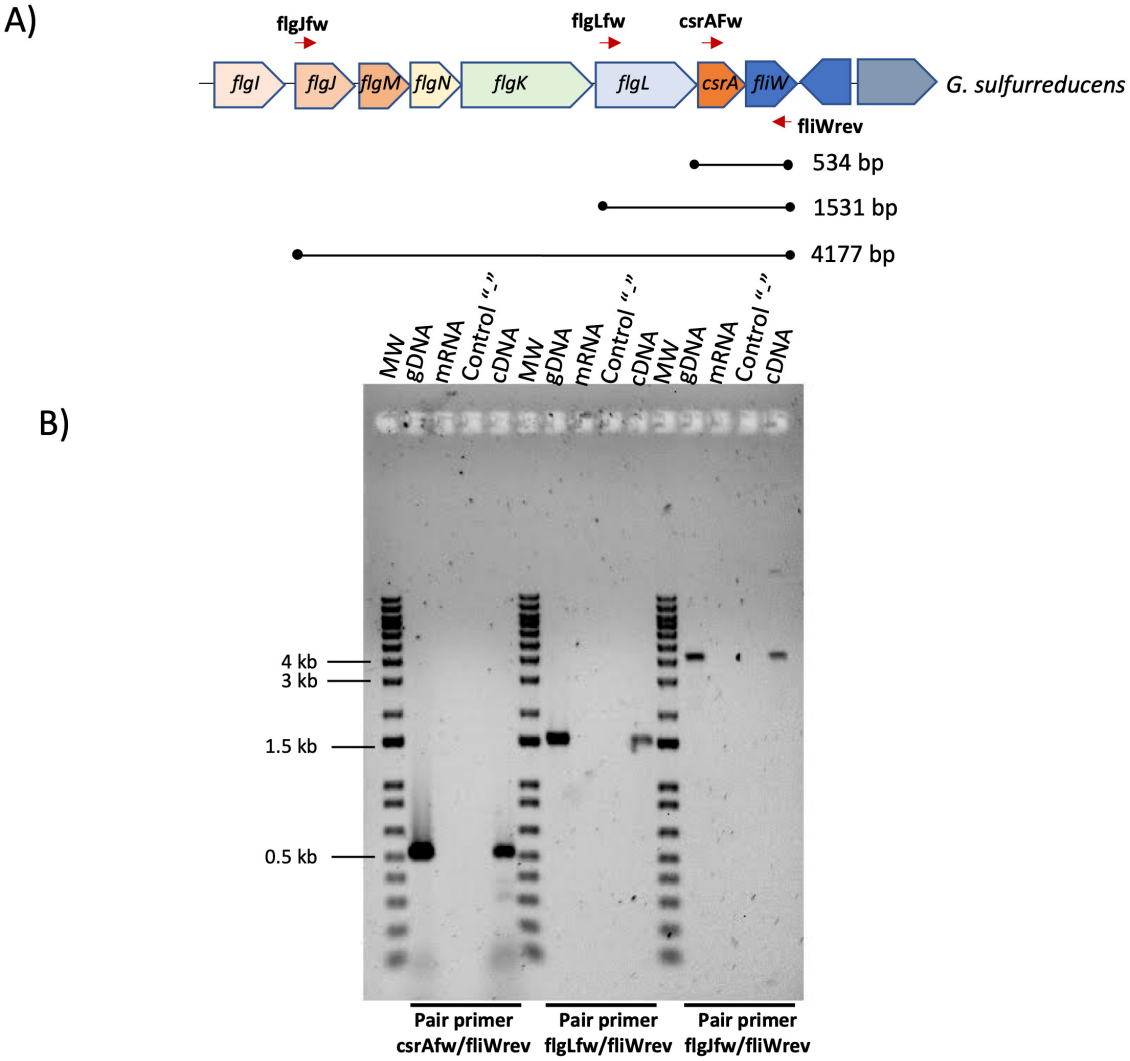
Determination of the *flgJMNKL-csrA-fliW* operon of *G. sulfurreducens*. **(A)** Gene map of the *flgJMNKL-csrA-fliW* operon. The thick arrows denote open reading frames. The lines between the closed circles below the arrows indicate the DNA regions amplified by PCR with the indicated primer pairs. The predicted molecular weight (MW) is shown. **(B)** RT-PCR was performed on total RNA isolated from *G. sulfurreducens* DL1 strain grown with acetate-fumarate. PCR was performed without a template (control “–”) or with mRNA, genomic DNA (gDNA), or cDNA as a template. As a MW we used 1 kb Plus DNA ladder (Invitrogen). Bottom from gel we indicated the pair primer used in each PCR reaction.

To determine if *csrA* is co-transcribed with the *flgJMNKL-csrA-fliW* operon, total RNA isolated from DL1 and a primer corresponding to the 3′ end of *fliW* were used in a reverse transcription experiment to produce cDNA. This cDNA and primers corresponding to the 5′ end of *csrA, flgL, flgJ* and the 3′ end of *fliW* were used in a PCR that produced a DNA fragments of around 534, 1531, and 4,177-bp, respectively, indicating that *csrA* is transcribed with the *flgJMNKL-csrA-fliW* operon (Figures 2A, B).

### 3.2 Effect of Δ*csrA* mutation in growth and Fe(III) reduction

**Figure 3:**
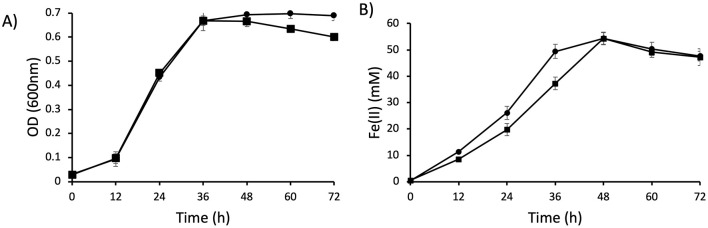
Growth and Fe(III) reduction of Δ*csrA* mutant. **(A)** Growth of DL1 and Δ*csrA* in NBAF (acetate-fumarate) medium. **(B)** Soluble Fe(III) reduction. Lines with square is DL1 and lines with circle are Δ*csrA* strain.

To characterize the role of CsrA in *G. sulfurreducens*, we constructed the Δ*csrA* mutant strain using the scarlees gene deletion system, as described previously (Chan et al., 2015). The Δ*csrA* mutant strain growth is similar to the wild type strain in the presence of acetate-fumarate (Figure 3A). In addition, the Δ*csrA* mutant strain reduces soluble Fe(III) 1.5-fold more than the wild type strain (Figure 3B). These data suggest that CsrA may negatively regulate genes encoding proteins involved in electron transport.

To confirm that the phenotype observed in Δ*csrA* strain related to growth and Fe(III) reduction was due to inactivation of the *csrA* gene, we also performed the complementation of this mutant strain. The plasmids pRG5.1 or pRG5.1-RRflg-*csrA* were transformed into the Δ*csrA* strain, as expected, the *csrA* strain with plasmids pRG5.1 or pRG5.1-RRflg-*csrA* showed a slight growth delay. We assumed that this effect may be a result of metabolic load, a phenotype previously observed in complementation strains in *G. sulfurreducens* (Juarez et al., 2009; Andrade et al., 2021; Hernández-Eligio et al., 2020) (Supplementary Figure 5).

### 3.3 *CsrA* negatively controls biofilm formation

**Figure 4:**
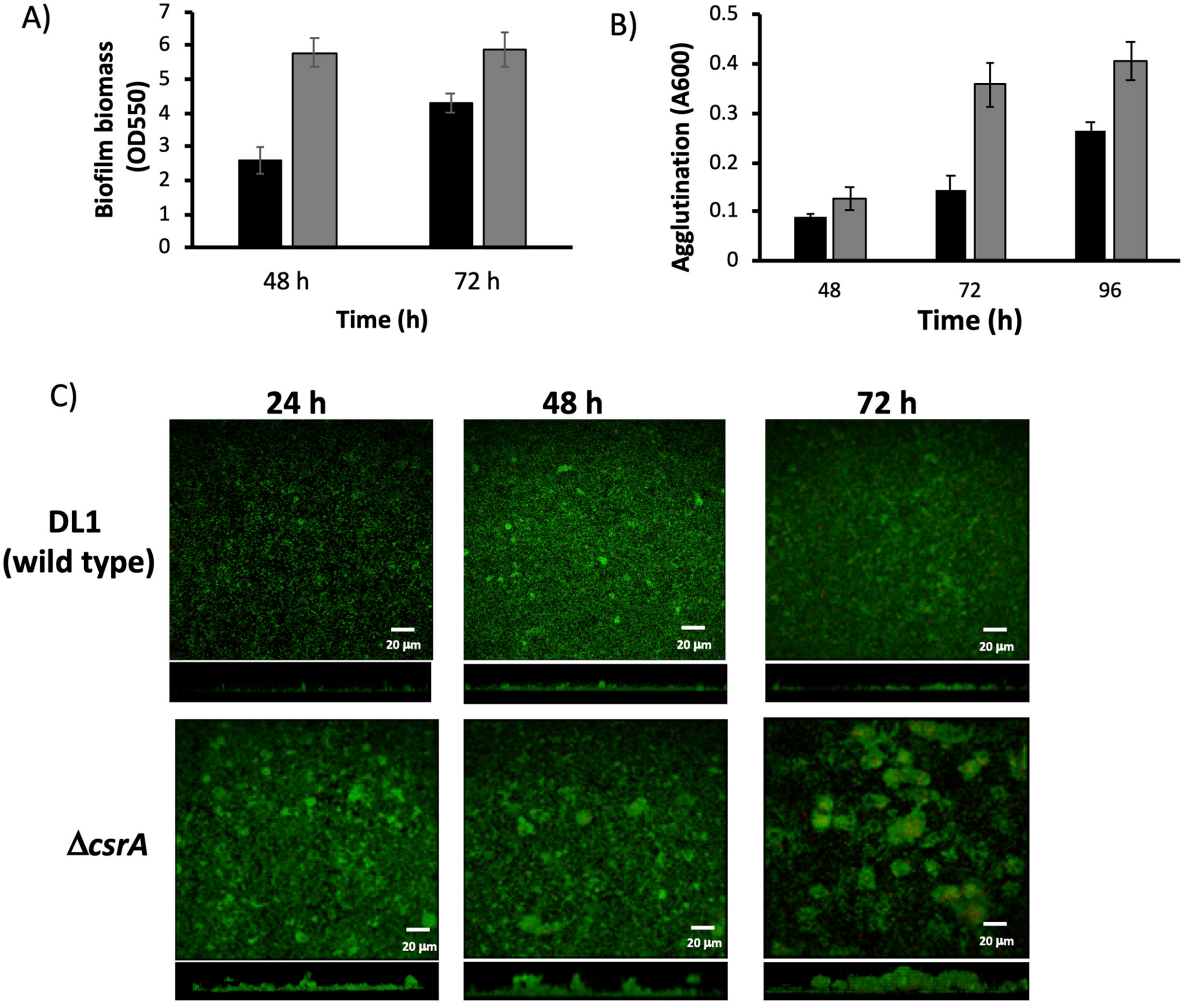
Characterization of biofilms and agglutination phenotypes of Δ*csrA* and wild type biofilms. **(A)** Biofilm biomass assessment by crystal violet of Δ*csrA* and DL1 strains. **(B)** Agglutination assay of Δ*csrA* and DL1 strains. In **(A, B)** the cells were grown with acetate-fumarate at 25°C for 48 and 72 h on the glass surface. Black bars are DL1 and gray bars are Δ*csrA*. **(C)** CLSM images of DL1 and Δ*csrA* biofilms grown on FTO-electrode in acetate-fumarate medium. The top and bottom panels illustrate the top and side view projections generated by CLMS of DL1 and Δ*csrA* biofilms at 24, 48, and 72 h. Live and dead cells are indicated in green and red, respectively. Red spots correspond to dead cells.

In *E. coli*, CsrA controls biofilm formation by controlling the expression of *pgaABCD* genes (Wang et al., 2005). To determine whether the CsrA regulator is involved in the biofilm formation regulation, first we performed crystal violet staining assays in order to quantify the biofilm formation by Δ*csrA* and wild type strains at two different time periods. Deletion of CsrA increases the biofilm 2-fold more than the wild type at 48 h of incubation (Figure 4A). Also at 72 h, biofilm formation in Δ*csrA* strain is thicker than wild type strain. These results could indicate that CsrA is involved in biofilm formation in *G. sulfurreducens*.

*In G. sulfurreducens*, the agglutination phenotype is related to pili production (Reguera et al., 2007). To determine if CsrA is involved in pili regulation, the agglutination phenotype in acetate-fumarate was determined at 25°C for 48, 72, and 96 h incubation periods in Δ*csrA* and wild type strains (Figure 4B). At 48 h, the Δ*csrA* strain agglutinated 0.4-fold more than the wild type, and at 72 and 96 h, the agglutination was increased 2.4- and 1.5-fold higher in the Δ*csrA* than the wild-type strain, respectively, suggesting that CsrA also regulates pilus formation, probably by controlling the translation of the *pilA* gene.

### 3.4 CLSM analysis

In previous work from our group, we have used Fluorine-doped tin oxide (FTO) electrodes to characterize the structure and development of biofilms of wild type and mutant strains of *G. sulfurreducens* using CLSM (Huerta-Miranda et al., 2019; Hernández-Eligio et al., 2022; Jaramillo-Rodríguez et al., 2023; Rodríguez-Torres et al., 2024). FTO electrodes are an excellent support material for the development of *G. sulfurreducens* biofilms as it promotes the development of thick and electroactive biofilms. To characterize the structure and development of biofilms on FTO electrodes by wild type and Δ*csrA* strains, biofilms were grown in the presence of acetate-fumarate and then analyzed by CLSM at three time periods (24, 48, and 72 h). Figure 4C (top) shows the CLSM images of the wild type biofilm at 24, 48, and 72 h. The wild type biofilm shows a slow development, with an increase in biomass and thickness at 48 h, which remains constant until 72 h. In the last time period, some dead cells (red dots) were observed in the biofilms of the wild type strain, which is consistent with a slight decrease in cell viability (Table 1). On the other hand, Figure 4C (bottom) shows the biofilm of the Δ*csrA* strain analyzed at the same incubation periods. The thickness of the biofilm of Δ*csrA* increases to almost double that of the wild type biofilm at 24 h, becoming up to 2.2-fold thicker than the wild type biofilm at 48 and 72 h. The biofilms of the Δ*csrA* strain contain a high percentage of viability, reaching >95% (Table 1).

**Table 1:** Biofilms parameters: thickness, roughness coefficient, substrate coverage, and viability.

		**Strain**	
	**Growth time/h**	**DL1**	**Δ*csrA***
Thickness/μm	24	6.88 ± 0.23	12.48 ± 1.35
	48	8.48 ± 1.35	18.66 ± 4.81
	72	9.75 ± 2.01	21.92 ± 13.24
Roughness coefficient	24	1.93 ± 0.03	1.56 ± 0.12
	48	1.59 ± 0.02	1.48 ± 0.11
	72	1.45 ± 0.16	1.65 ± 0.07
Substrate coverage %	24	4.79 ± 1.34	25.57 ± 8.89
	48	22.10 ± 2.27	29.95 ± 2.96
	72	21.05 ± 1.42	22.74 ± 4.31
Viability %	24	96.29 ± 1.82	97.47 ± 0.35
	48	97.19 ± 0.03	97.07 ± 2.92
	72	92.26 ± 5.18	95.83 ± 1.69

Values obtained from CLSM analysis.

The other parameters analyzed by CLSM are presented in Table 1. The roughness coefficient, a variable related to the thickness and heterogeneity of the biofilms, is similar in the biofilms of both strains, indicating that the biofilms are heterogeneous. In the biofilms of the Δ*csrA* strain, cell aggregates are observed at 48 h, increasing in thickness until 72 h, resulting in a maximum roughness coefficient of 1.6 in the same period. Finally, the substrate coverage value in wild-type biofilms is low at 24 h and increases at 48 and 72 h, while the substrate coverage values of Δ*csrA* biofilms are high in all three time periods, suggesting that the Δ*csrA* strain is more efficient in the electrode colonization process.

### 3.5 Transcriptome of Δ*csrA* biofilm formed in glass support

**Figure 5:**
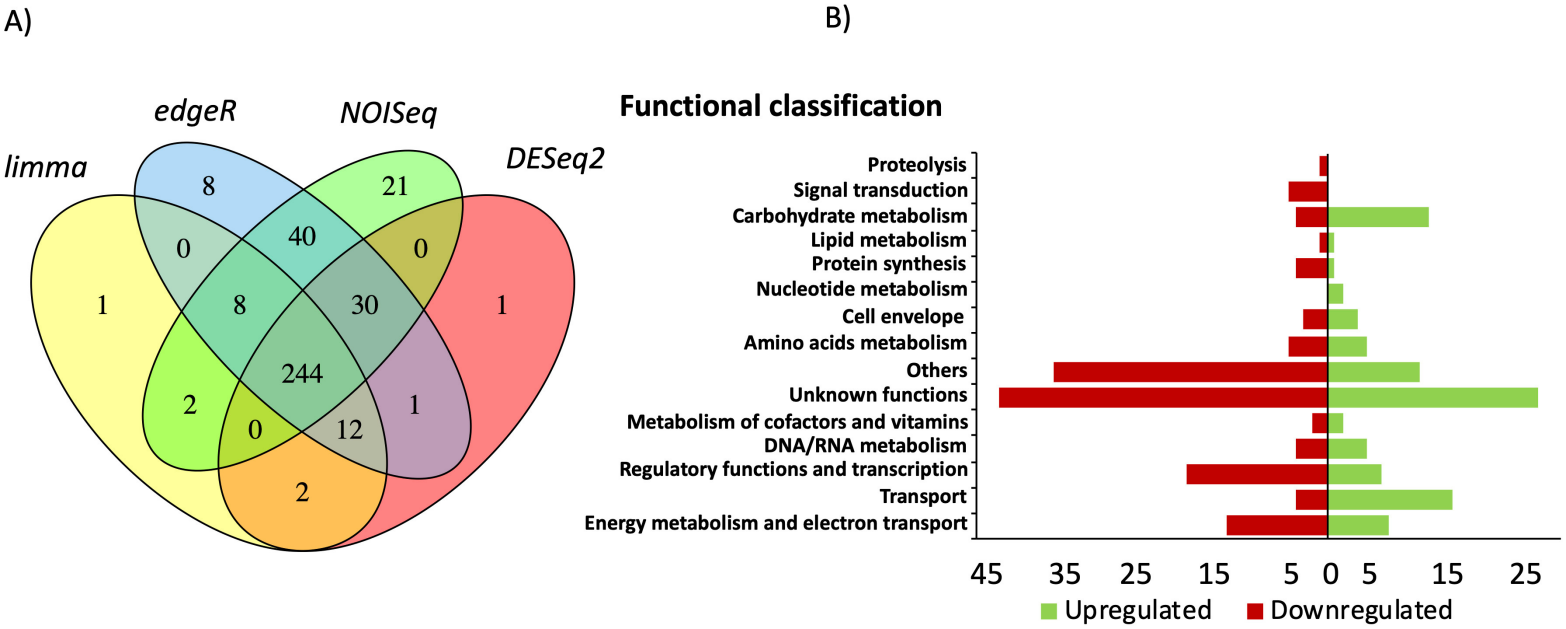
Transcriptional analysis results in Δ*csrA* vs. wild-type biofilms grown in glass. **(A)** Venn diagram of differentially expressed genes identified using limma, edgeR, NOISeq, and DESeq2 statistical methods. **(B)** Functional classification of the genes that were differentially expressed in Δ*csrA* vs. wild-type biofilms.

To investigate the role of CsrA as a regulator of biofilm formation in *G. sulfurreducens*, we performed RNA-seq transcriptome analysis of Δ*csrA* and wild type biofilms grown for 48 h. A total of 244 genes (103 upregulated and 141 downregulated) showed significant differential expression in Δ*crsA* biofilms to wild type biofilms, suggesting that CsrA may act as either an activator or a repressor in *G. sulfurreducens* (Supplementary Table 2). These genes were grouped into 15 functional categories (Figure 5). The functional categories with a higher number of differentially expressed genes in Δ*csrA* biofilms are: unknown functions (69), others (47), regulatory functions (25), energy and metabolism (21), transport (20), and carbohydrate metabolism (17). Principal component analysis (PCA) highlighted distinct clustering of replicate strains, with the most significant variance (94%) between Δ*csrA* and DL1 strain being captured by the first principal component (PC1) (Supplementary Figure 2A). Only genes that showed differential expression (*p* < 0.05 and logFC > 1.5) by the four statistical methods evaluated in this study, limma, DESeq2, edgeR, and NOISeq, were included. A total of 244 genes (103 upregulated and 141 downregulated) showed significant differential expression in Δ*crsA* biofilms to wild type biofilms, suggesting that CsrA may act as either an activator or a repressor in *G. sulfurreducens* (Supplementary Table 2). These genes were grouped into 15 functional categories (Figure 5). The functional categories with a higher number of differentially expressed genes in Δ*csrA* biofilms are: unknown functions (69), others (47), regulatory functions (25), energy and metabolism (21), transport (20), and carbohydrate metabolism (17).

### 3.6 RT-qPCR of selected genes with changes in RNA-seq

To validate the data obtained in the RNA-seq analysis, we quantified the expression of some differentially expressed genes by RT-qPCR, selected genes related to transport (*feoB*-2, GSU0972), cytochromes c (GSU3274, *ppcB, omcH*), transcriptional regulators (GSU0470, *gnfR*, GSU0018), diguanylate cyclases (GSU1554, GSU2044), hypothetical proteins (GSU3409, GSU0597, GSU3410), carbohydrate metabolism (*rmlB*, GSU1970), nucleotide metabolism (GSU2236), cell envelope (GSU1963), amino acids metabolism (GSU3142), proteolysis (GSU1944), and others (GSU3014, GSU0490, GSU1496) (Table 2).

**Table 2:** RT-qPCR validation of differentially expressed genes elucidated by RNA-seq.

**Locus ID**	**Description**	**Functional category**	**Avg Δ*csrA*/Avg DL1**
GSU2366	*rmlB*, dTDP-glucose 4,6-dehydratase	Carbohydrate metabolism	1.5 ± 0.05
GSU3268	*feoB*-2, ferrous iron transport protein B	Transport	1.64 ± 0.25
GSU3274	cytochrome c, 1 heme-binding site	Energy metabolism and electron transport	1.87 ± 0.02
GSU2236	*relA*, GTP diphosphokinase	Nucleotide metabolism	3.37 ± 1.87
GSU1963	Undecaprenyl-diphospho-oligosaccharide flippase	Cell envelope	4.73 ± 0.16
GSU3142	*aroG-2*, 3-deoxy-7-phosphoheptulonate synthase	Amino acids metabolism	3.57 ± 0.09
GSU0972	ATPase, AAA family	Transport	6.97 ± 2.05
GSU1970	*neuB*, N-acetylneuraminate synthase	Carbohydrate metabolism	2.28 ± 0.53
GSU0470	σ54-dependent transcriptional response regulator	Regulatory functions and transcription	1.87 ± 0.46
GSU0364	*ppcB*, cytochrome c	Energy metabolism and electron transport	0.04 ± 0.04
GSU1496	*pilA-N*, geopilin domain 1 protein	Others	0.01 ± 0.01
GSU1944	PEP motif-containing protein	Proteolysis	0.02 ± 0.03
GSU3014	metal-dependent phosphohydrolase	Others	0.04 ± 0.04
GSU0490	*ato-1*, succinyl:acetate coenzyme A transferase	Others	0.03 ± 0.04
GSU2883	*omcH*, cytochrome c	Energy metabolism and electron transport	0.26 ± 0.27
GSU2822	*gnfR*, response regulator	Regulatory functions and transcription	0.06 ± 0.04
GSU3409	Hypothetical protein	Unknown functions	0.61 ± 0.04
GSU0597	Hypothetical protein	Unknown functions	0.15 ± 0.09
GSU3410	Hypothetical protein	Unknown functions	0.19 ± 0.07
GSU0018	Transcriptional regulator, GntR family/aminotransferase class-I	Regulatory functions and transcription	0.35 ± 0.27
GSU1554	Diguanylate cyclase	Signal transduction	0.03 ± 0.02
GSU2044	Sensor diguanylate cyclase/phosphodiesterase	Signal transduction	0.25 ± 0.18

High expression of GSU3274, GSU2236, GSU3142, and GSU0470, as well as the genes encoding the transporters GSU0972 and *feoB*-2, and the carbohydrate-synthesis-related genes GSU1970, GSU1963 and *rmlB* was verified in Δ*csrA* biofilms. While we found down expression of genes encoding the hypothetical proteins GSU3409, GSU0597, GSU3410. In addition, the genes encoding the putative diguanylate cyclases GSU1554 and GSU2044, as well as the cytochromes *ppcB* and *omcH* are downregulated in Δ*csrA* biofilms. Finally, the decreased expression of *pilA*, GSU1944, GSU3014, GSU0490, and the transcriptional regulatory genes GSU2822 and GSU0018 were confirmed in the Δ*csrA* biofilms (Table 2). Although the expression values in the RNA-seq dataset are different from the RT-qPCR results, the expression trend is the same, suggesting a high reliability of the RNA-seq results.

### 3.7 Current generation by Δ*csrA* strain

**Figure 6:**
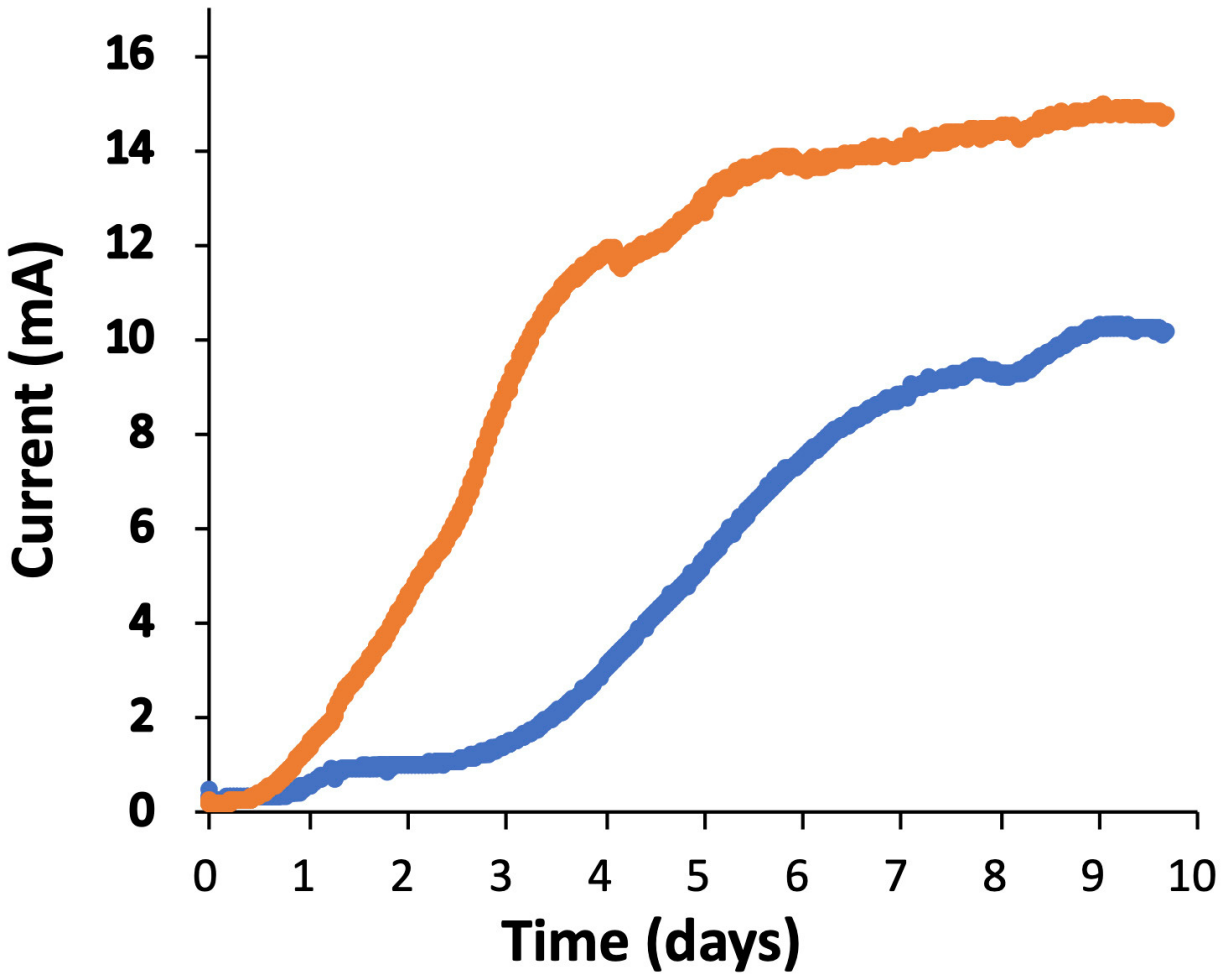
Current production of Δ*csrA* and DL1 strains. The blue and orange lines represent the current production of DL1 and Δ*csrA* strains, respectively, as a function time. The results presented in the graphic are representative time courses for three replicates for each treatment.

To determine the current production of the Δ*csrA* mutant strain, cells were grown in two-chamber MFC. Cells were grown on acetate-fumarate and an inoculum harvested during the exponential phase was transferred anaerobically to the anode chamber with a graphite electrode. A constant flow of acetate was maintained at the anode after the current was started. As shown in Figure 6, the DL1 strain began to generate current at day 3 and reached a plateau at day 7. The DL1 reached a maximum current of 10 mA after 10 days. On the other hand, the Δ*csrA* strain started to generate current on day 1 and reached a maximum of 14.7 mA on day 9. After both strains reached the current production plateau, the Δ*csrA* strain produced 45–50% more current than the DL1 strain. The increase in current by Δ*csrA* strain may be due to the fact that several genes involved in extracellular electron transfer *c*-type cytochromes and those encoding enzymes involved in exopolysaccharide synthesis are upregulated in the biofilms. To investigate this possibility, electrodes were removed at the end of MFC operation and biofilms were collected for transcriptome analysis by RNA-seq.

### 3.8 Transcriptome of Δ*csrA* biofilm grown in graphite electrode

**Figure 7:**
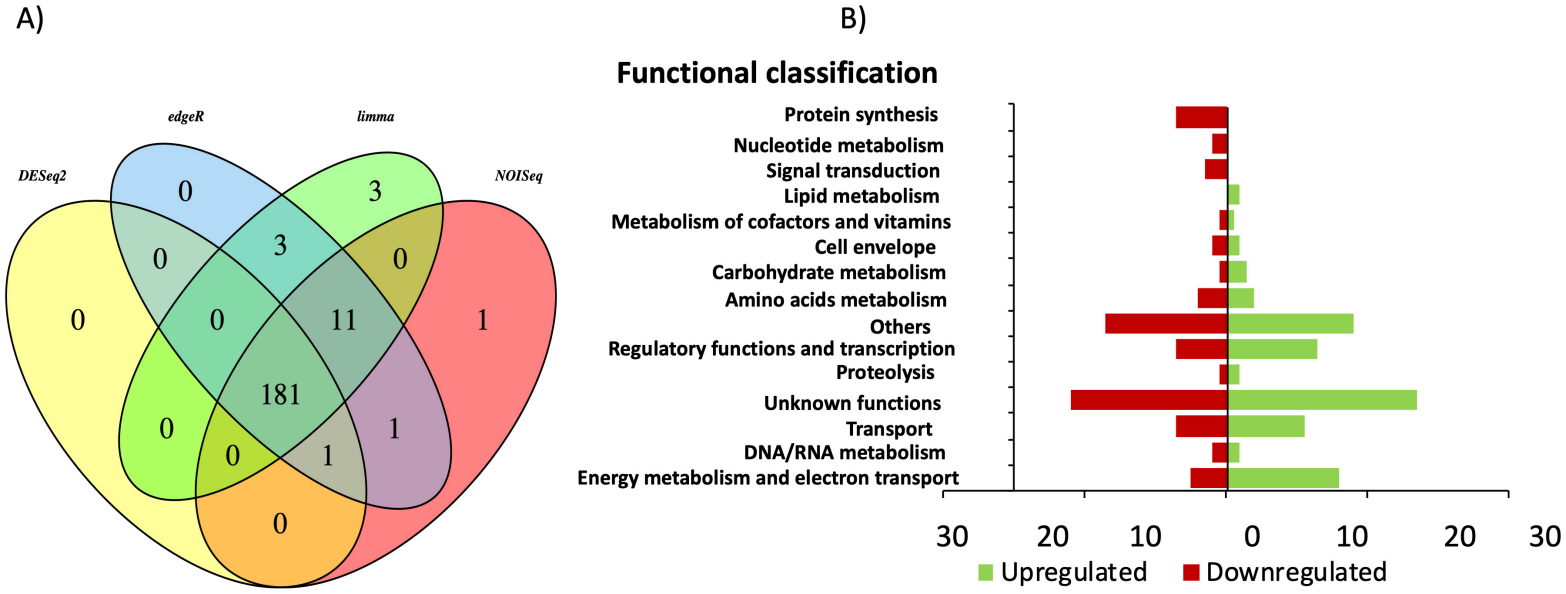
Differential gene expression in Δ*csrA* vs. wild-type biofilms grown in MFC. **(A)** Venn diagram of DE identified using limma, edgeR, NOISeq, and DEseq2 methods. **(B)** Functional overview of genes that were DE in the Δ*csrA* biofilm.

To determine the transcriptomic response of Δ*csrA* biofilm producing current in MFC, RNA was extracted from biofilms grown on graphite electrodes, and RNA-seq analysis was performed. Principal component analysis (PCA) showed distinct clustering of replicate strains, with the higher significant variance (96%) between Δ*csrA* and DL1 strain being determined by the first principal component (PC1) (Supplementary Figure 2B). Under these conditions, we found 181 DE genes (100 upregulated and 81 downregulated). All DE genes were grouped into 15 categories, including: “energy metabolism and electron transport,” “DNA/RNA metabolism,” “transport,” “unknown functions,” “proteolysis,” “regulatory functions and transcription,” “others,” carbohydrate metabolism,” “cell envelope,” “metabolism of cofactors and vitamins,” “lipid metabolism,” “signal transduction,” “nucleotide metabolism,” and “protein synthesis” (Figure 7). The five categories with the most DE genes are unknown function (49), others (35), energy metabolism and electron transport (21), regulatory function and transcription (20), and transport (18) (Supplementary Table 3).

Of the 181 DE genes in the transcriptome of Δ*csrA* biofilms grown on graphite electrodes, 68 genes are also show expression changes in biofilms grown on glass, of which 62 genes show the same regulation type (34 upregulated and 28 downregulated), while six show counter-regulation (Table 3). Among the DE genes in both RNA-seq datasets are those encoding a hypothetical protein, diguanylate cyclases, transcriptional regulators, transporters, glycosyl transferases, hydrogenases, membrane proteins, pili-related proteins, among others (Table 3).

**Table 3:** List of differentially expressed genes in Δ*csrA* compared with the DL1 strain in both RNA-seq analysis.

**Regulation**	**Locus tag**	**Name**	**Glass (Log2FC)**	**MFC (Log2FC)**
Upregulated	GSU0470	Sigma-54 dependent response regulator	2.44	2.85
	GSU0471	Sensor histidine kinase	2.66	2.73
	GSU0784	hybB, nickel-dependent hydrogenase	1.74	2.74
	GSU0785	hybL, nickel-dependent hydrogenase, large subunit	1.98	4.27
	GSU0786	hybP, hydrogenase maturation protease	1.63	3.44
	GSU0972	ATPase, AAA family	2.72	2.98
	GSU0973	Hypothetical protein	2.57	2.46
	GSU0974	Hypothetical protein	2.67	2.97
	GSU0975	phage tail sheath protein, putative	2.50	2.54
	GSU0976	Hypothetical protein	2.46	2.23
	GSU0977	Hypothetical protein	3.10	2.72
	GSU0978	Hypothetical protein	2.28	2.01
	GSU0980	Hypothetical protein	2.71	1.80
	GSU0981	Hypothetical protein	2.23	2.61
	GSU0982	Phage protein D, putative	2.64	1.95
	GSU0983	Phage tail spike protein, putative	2.52	1.59
	GSU0985	PAAR motif-containing protein	2.94	2.34
	GSU0986	Tail lysozyme, putative	3.03	2.49
	GSU0987	Hypothetical protein	3.84	2.79
	GSU0988	Hypothetical protein	3.10	2.35
	GSU0989	NHL repeat domain protein	3.39	3.28
	GSU0990	Hypothetical protein	3.58	3.51
	GSU0991	Glycosyltransferase, ExpC-like family	3.57	3.14
	GSU0992	Hypothetical protein	3.68	2.97
	GSU1153	Outer membrane protein, OMP85 family	2.38	2.13
	GSU1154	Surface repeat protein, putative	1.82	2.05
	GSU1380	feoB-1, ferrous iron transport protein B	3.18	1.60
	GSU1962	Glycosyl transferase	3.34	2.47
	GSU2782	Efflux pump, RND family, AcrB/AcrD/AcrF family	1.70	1.78
	GSU2801	Cytochrome c	1.78	1.92
	GSU2967	Ferritin-like domain protein	1.57	4.53
	GSU2968	Hypothetical protein	2.30	5.18
	GSU3141	Hypothetical protein	3.28	2.62
	GSU3142	aroG-2, 3-deoxy-7-phosphoheptulonate synthase	3.57	2.14
Downregulated	GSU0071	Hypothetical protein	−3.87	−2.97
	GSU0081	Hypothetical protein	−2.02	−1.59
	GSU0216	Hypothetical protein	−2.85	−1.90
	GSU0444	Hypothetical protein	−3.41	−1.67
	GSU0547	mutS-2, DNA mismatch repair ATPase MutS-2	−3.17	−1.98
	GSU0548	Radical SAM domain protein	−3.35	−1.93
	GSU0884	Radical SAM domain protein	−2.26	−1.91
	GSU0919	Hypothetical protein	−1.70	−1.89
	GSU1037	Response receiver-modulated diguanylate cyclase/phosphodiesterase	−2.03	−3.61
	GSU1176	frdC, succinate dehydrogenase/fumarate reductase	−2.45	−1.74
	GSU1395	Hypothetical protein	−2.00	−3.25
	GSU1496	pilA-N, geopilin domain 1 protein	−3.06	−2.56
	GSU1497	pilA-C, geopilin domain 2 protein	−3.31	−2.50
	GSU1669	Hypothetical protein	−2.58	−1.52
	GSU1746	ihfB-1, integration host factor, beta subunit	−1.77	−1.69
	GSU1877	oxidoreductase, 2-nitropropane dioxygenase family	−3.57	−2.45
	GSU2044	Sensor diguanylate cyclase/phosphodiesterase	−2.81	−1.85
	GSU2487	cpkA, carbamate kinase	−1.58	−1.92
	GSU2585	Hypothetical protein	−2.80	−1.64
	GSU2614	recJ, single-stranded-DNA-specific exonuclease	−2.58	−2.53
	GSU2641	PATAN domain GTPase-activating protein, putative	−2.33	−2.46
	GSU2662	Membrane protein, putative	−3.72	−4.36
	GSU2663	Lipoprotein, putative	−4.70	−4.99
	GSU2896	Ankyrin repeat protein	−1.71	−1.74
	GSU2938	Hypothetical protein	−1.91	−1.71
	GSU3098	hisB, imidazoleglycerol-phosphate dehydratase	−1.58	−2.05
	GSU3304	ompJ, outer membrane channel OmpJ	−1.68	−1.52
	GSU3629	Hypothetical protein	−3.25	−2.45
Counterregulated	GSU0012	hemY, protoporphyrinogen oxidase	−2.49	1.55
	GSU0013	Winged helix-turn-helix transcriptional regulator, MarR family	−2.50	2.01
	GSU0182	Murein lipoprotein	−1.96	1.64
	GSU1943	Putative exosortase substrate	−3.37	1.50
	GSU1944	Putative exosortase substrate	−4.37	1.71
	GSU2135	efflux pump, RND family, inner membrane protein	3.49	−3.05

### 3.9 Differentially expressed genes only in MFC

Among the genes that increase their expression in biofilms of the Δ*csrA* strain in MFC are those encoding proteins related to energy metabolism and electron transport. These include the genes encoding the cytochromes OmcX, GSU0702, OmcM, GSU2513, and GSU2808 (Supplementary Table 4). The cytochrome OmcX, with a predicted localization in the periplasm, is also upregulated in biofilms grown in graphite electrodes poised at −0.17 V to biofilms poised at the same potential in the presence of formate (“medium pathway,” called by the authors), and its presence is increased in response to the presence of hydrogen as electron donor (Howley et al., 2023; Mollaei et al., 2021). The cytochrome GSU0702, predicted to be extracellular, is upregulated in Δ*gsu1771* biofilms of graphite electrodes and wild-type “medium pathway” biofilms (Jaramillo-Rodríguez et al., 2023; Howley et al., 2023). On the other hand, cytochrome OmcM is also upregulated in biofilms grown on graphite electrodes of strain Δ*gsu1771*. This cytochrome is related to the reduction of Fe(III) oxides since its absence negatively affects Fe(III) reduction (Jaramillo-Rodríguez et al., 2023; Aklujkar et al., 2013). The genes encoding the cytochromes GSU2513 and GSU2808 were found to be upregulated under Pd(II) reducing conditions (Hernández-Eligio et al., 2020). In addition, GSU2808 was significantly upregulated in the formate −0.17 V biofilm compared to the acetate anode biofilm conditions (Howley et al., 2023). Among the genes encoding cytochromes that were downregulated were *omcT, omcS*, and GSU3214. It is known that *omcT* and *omcS* are organized in an operon and that OmcS is involved in the reduction of Fe(III) oxides, but the two cytochromes are not relevant for electrode respiration (Mehta et al., 2005). The GSU3214 gene was found to be upregulated under conditions of Fe(III) and Mn(IV) oxide reduction (Aklujkar et al., 2013).

The *hybS* and *hybA* genes were found to be upregulated. HybS and HybA encode subunits of the Ni-Fe hydrogenase HyB. The HyB hydrogenase is involved in respiration with hydrogen as an electron donor and fumarate, AQDS, and Fe(III)-citrate as acceptors (Coppi, 2005). It is proposed that HyB accepts electrons from hydrogen and transfers them to the menaquinone pool where they are redistributed to various reductases (Coppi, 2005). Under electron donor format conditions all Hyb hydrogenase subunits are increased compared to acetate conditions (Mollaei et al., 2021).

The genes GSU2096, GSU2097, and GSU2098 are upregulated in Δ*csrA* biofilms grown in MFC. In *G. sulfurreducens*, GSU2098 encodes a putative monofunctional carbon monoxide dehydrogenase (CODH) that may be involved in carbon monoxide metabolism in *G. sulfurreducens* (Geelhoed et al., 2015). GSU2097 putatively encodes a homolog of CooC, an accessory protein involved in ATP-dependent Ni-insertion into CODH. *GSU2096* encodes an iron-sulfur cluster-binding protein containing binding sites for two [4Fe-4S] clusters. GSU2096 is proposed to mediate electron transfer from CooS via the Fe-S clusters (Geelhoed et al., 2015; Singer et al., 2006).

On the other hand, among the genes encoding transporters found upregulated in Δ*csrA* biofilms grown in MFC are the transporters GSU0433 (*tssH*), GSU0575, GSU0706, GSU0707, GSU1279, GSU2778, and GSU2781. GSU0433 is part of a type VI secretion system and was reported to be overregulated under DIET vs. QUIET conditions in co-cultures of *G. sulfurreducens* and *G. metallireducens* (Smith et al., 2023). Under these conditions, it is proposed that overexpression of the type VI secretion system affects the establishment of associations between different Geobacter species. On the other hand, the proteins GSU0706 and GSU0707 are proposed to be quaternary ammonium transporters, while GSU0575 is a putative peptide transporter and GSU2781 is an ABC-type nickel transporter.

Among the regulatory genes with transcriptional changes in Δ*csrA* biofilms are several that encode putative histidine kinases and response regulators. These are the genes GSU1148, GSU1264, GSU3261, GSU3419, which are also upregulated in Δ*gsu1771* biofilms (Jaramillo-Rodríguez et al., 2023). The genes GSU0475, GSU1265, and the regulator GSU2214 are involved in chemotaxis. The GSU1999 gene, which encodes a homolog of the Hfq protein, is also upregulated.

### 3.10 Putative binding sites of CsrA in *G. sulfurreducens*

In γ-proteobacteria, CsrA binds directly to a site-specific sequence in the 5′-UTR region of mRNAs target. The CsrA-binding sequence is located in a hairpin structure, and the GGA motif is conserved in the loop (Vakulskas et al., 2015). To assess whether there are putative CsrA binding sites in the 5-UTR region of the genes that changed their expression in both transcriptome analyses (244 genes with DE in biofilms grown in glass and 113 with DE only in biofilms grown in MFC), we performed an *in-silico* analysis using Multiple Em for Motif Elicitation (MEME) (Bailey et al., 2015). In the MEME analysis, 150 nucleotides upstream of the translation start of the genes that changed their expression were analyzed. Of the 357 genes analyzed, 76 contain the consensus sequence VVAAGGAGRV (where V is A, C, or G, and R is G or A) in their 5′-UTR region (Supplementary Table 5). Of the 76 putative binding sites, 60 are located between −10 and −1, and 16 are located between −11 and −135 for start translation of the target gene. The putative CsrA binding sites located at −10 of the translation start site of the mRNA target suggest that these are CsrA binding sites and that CsrA may directly regulate their translation. RNA-protein interaction assays, together with substitutions in the putative binding site, would be expected to confirm this hypothesis.

In γ-proteobacteria, the activity of CsrA is controlled by small sRNAs that are members of the CsrB/CsrC family. In many bacteria where CsrA has been studied, the small sRNAs that regulate its function have not been identified because some RNAs are specific or not conserved among bacterial genomes. In a recent paper, using a software called InvenireSRNA, which combines sequence and structure with machine learning, predicted the presence of 3 genes encoding putative CsrB/CsrC family sRNAs in the genome of *G. sulfurreducens* strain KN400 (Fakhry et al., 2017). These three putative genes are conserved in the PCA strain genome, two are encoded in the antisense of the GSU0072 and GSU2675 genes and one in the intergenic region of the GSU1100 and *phoR* genes. The sequence of the predicted sRNAs (sRNA-1, sRNA-2, and sRNA-3) and their secondary structure generated by RNAfold (Lorenz et al., 2011) are shown in Supplementary Figure 6. Nucleotide sequence analysis reveals that the predicted sRNAs contain multiple GGA motifs and are located within regions that are conserved relative to the consensus identified by MEME analysis (Supplementary Figure 7). The possibility that these genes encode sRNAs that regulate CsrA function will be explored in the future.

On the other hand, in the Gram-positive bacteria *Bacillus subtilis* and *Campylobacter jejuni* CsrA is involved in flagellar regulation, and its function is antagonized by the FliW protein (El Abbar et al., 2019; Oshiro et al., 2020). In both bacteria, CsrA contains a conserved N55 residue at the C-terminal, which is critical for interaction with FliW (Figure 1) (Oshiro et al., 2020; Bogacz et al., 2021). Similarly, CsrA from *G. sulfurreducens* has a conserved N55 residue in its C-terminal, suggesting that FliW may be involved in the regulation of CsrA function, together with the fact that its genes are organized in an operon (Figure 2). The possibility that the CsrA function of *G. sulfurreducens* is regulated by both the CsrB/CsrC family sRNAs and the FliW protein highlights the relevance of this post-transcriptional regulator in the control of biofilm formation, EET, and other cellular processes.

## 4 Conclusion

CsrA is a global post-transcriptional regulator in *Geobacter sulfurreducens* involved in several cellular functions including biofilm formation. Δ*csrA* mutant produces up to twice the biofilm thickness than WT strain, and the global scale response revealed that 244 genes were differentially expressed in the biofilm developed in glass support, extracellular electron transfer, exopolysaccharide synthesis, c-di-GMP synthesis and degradation were the metabolic functions affected. Furthermore, changes in gene expression during current production in MFC, demonstrated that in *G. sulfurreducens* CsrA is involved in the expression of some diguanylate cyclases, transcriptional regulators, transporters, glycosyl transferases, hydrogenases, membrane proteins and type-4 pili during current production, all these metabolic changes resulting in 40% higher current production than WT strain. More detailed information about the metabolic processes regulated by CsrA in *G. sulfurreducens* could be obtained by future proteomic studies. This is the first report describing the role of CsrA in the regulation of extracellular electron transfer and biofilm in a member of the δ-proteobacteria.

## Data Availability

The original contributions presented in the study are publicly available. This data can be found here: https://www.ncbi.nlm.nih.gov/, accession number GSE282747.
