# Supplementary material for: The role of CsrA in controls the extracellular electron transfer and biofilm production in Geobacter sulfurreducens
Source: Front Microbiol. 2025 Mar 11;16:1534446. doi: 10.3389/fmicb.2025.1534446 (PMC11934962; doi:10.3389/fmicb.2025.1534446)
Supplement: Supplementary file 1 [file Table_1.docx]

**Supplementary Table 1** List of strains, plasmids, and oligonucleotides used in this work.

| **Name** | **Description** | **Reference** |
| --- | --- | --- |
| **Strains** |  |  |
| *Geobacter sulfurreducens* | |  |
| DL1 | Wild type | Caccavo et al., 1994 |
| Δ*csrA* | DL1 with *csrA* mutation | This work |
| DL1/pRG5.1 | Wild type with pRG5.1 vector | This work |
| Δ*csrA*/pRG5.1 | Δ*csrA* with pRG5.1 vector | This work |
| Δ*csrA*/pRG5.1-RRflg-*csrA* | Δ*csrA* with pRG5.1-RRflg-*csrA* vector | This work |
| *Escherichia coli* | |  |
| S17-1 | *recA pro hsdR RP-4-2-Tc::Mu-Km::Tn7* | Simon et al., 1983 |
| DH5α | *F− endA1 glnV44 thi-1 recA1 relA1 gyrA96 deoR nupG purB20* | Invitrogen |
|  |  |  |
| **Plasmids** |  |  |
| pk18mobsacB | Plasmid suicide, for scarless deletions | Simon et al., 1983 |
| pk18mobsacB-csrAdel | pK18mobsacB with the flanking regions of *csrA* gene | This work |
| pRG5.1 | Bacterial expression vector, spectinomycin resistance | Kim et al., 2005 |
| pJET1.2 | Subcloning vector, ampicillin resistance | Thermo |
| pJET-RRflg | pJET1.2 with RRflg fragment | This work |
| pJET-RRflg-*csrA* | pJET-RRflg with *csrA* gene | This work |
| pRG5.1-RRflg-*csrA* | pRG5.1 with RRflg-*csrA* fusion | This work |
|  |  |  |
| **Oligonucleotides** | **5´→3´** |  |
| *for mutant construction* | |  |
| csrAFw1Bam | GACT**GGATCC**CCAGCAGATTCTCCACA | This work |
| csrARv2quim | CGTTATTACTCCTTCCCGTCCCCCCTACGAGAGGTAGTCA | This work |
| csrAFw3quim | TGACTACCTCTCGTAGGGGGGACGGGAAGGAGTAATAACG | This work |
| csrARv4Eco | GCAT**GAATTC**CCCGTCTTTCAGCTTTCATC | This work |
| *for RT-PCR* |  |  |
| fliWrev | TGCTCCCCTCCAGAACGAT | This work |
| flgLfw | ACGGCAAACCTGGTTCTCA | This work |
| flgJfw | ATGCGTACAAGTATGCCGAC | This work |
| csrAFw | AATTCATATGTTAGTACTGACCAG | This work |
| *for cloning* |  |  |
| RRflgEcoRIfw | AATTGAATTCATTCTCCAGGCAATCAAGG | This work |
| RRflgNdeIrev | AATTCATATGATTCACCTATCGGCCAACT | This work |
| csrAFwNdeI | AATT*CATATG*TTAGTACTGACCAG | This work |
| csrAHindIIIrev | AATT*AAGCTT*TTACTCCTTCCCGTC | This work |
|  |  |  |
| *for RT-qPCR* |  |  |
| recA660f | GTGAAGGTGGTCAAGAACAAGGT | Holmes et al., 2006 |
| recA737r | GGAAATGCCCTCACCGTAGTAA | Holmes et al., 2006 |
| qPCRomcHfw | ATGGACGTGAATGGAAGGAG | Hernández-Eligio et al., 2020 |
| qPCRomcHrev | TGGCAGTCAGTACAGGTGGA | Hernández-Eligio et al., 2020 |
| qPCRppcBfw | CCACAAGAAACACCAGACGA | Hernández-Eligio et al., 2020 |
| qPCRppcBrev | CACCCCTTGCAGGACTTG | Hernández-Eligio et al., 2020 |
| qPCRpilAfw | AATTACCCCCATACCCCAAC | Hernández-Eligio et al., 2020 |
| qPCRpilArev | AGCAGCTCGATAAGGGTGAA | Hernández-Eligio et al., 2020 |
| qPCR2822Fw | CTTCCCGACGTCATCATTCT | Andrade et al., 2021 |
| qPCR2822Rev | CATCATAGCAGGCCGTCAG | Andrade et al., 2021 |
| qPCRaroG2fw | CAATGCCCAGAACTTTGCCC | Jaramillo-Rodriguez et al., 2023 |
| qPCRaroG2rv | TCATGACATACTCGGCGCTC | Jaramillo-Rodriguez et al., 2023 |
| qPCRato1fw | CATCGCCAACGCTGTAATCG | Jaramillo-Rodriguez et al., 2023 |
| qPCRato1rv | ATCGAACAGGTCGAGCATGG | Jaramillo-Rodriguez et al., 2023 |
| qPCR0972fw | AACTCACGTCGCTCATGGAC | Jaramillo-Rodriguez et al., 2023 |
| qPCR0972rv | ATGAACACGTCGGAGGGATG | Jaramillo-Rodriguez et al., 2023 |
| qPCR2366fw | TGACACCTTTTCCCCTTCCG | This work |
| qPCR2366rv | ATCCAGATTGATCACCCGGC | This work |
| qPCRGSU3268fw | ATGAACAGGGTGATGGGCAG | This work |
| qPCRGSU3268rv | TTGATCAGGGTCGACTTGCC | This work |
| qPCRGSU3274fw | GGGGAATATGTACCGGCATCT | This work |
| qPCRGSU3274rv | TTCCTTGGTAATGTCGGGCC | This work |
| qPCRGSU2236fw | GAACGCCAGGAACAGAAGGA | This work |
| qPCRGSU2236rv | ACAAGAACATCGTCGACCCC | This work |
| qPCRGSU1944fw | ATTGGCCAAGCATTCTGTCG | This work |
| qPCRGSU1944rv | AATGCGTCGAGATTGAGCCA | This work |
| qPCRGSU3014fw | CATTTCTGGCCGGCTTGTTC | This work |
| qPCRGSU3014rv | GCCCTTCATTGTAGCAACGC | This work |
| qPCRgsu1970fw | GTTTCTGAAGTACGTGGCGC | This work |
| qPCRgsu1970rv | CCTGATCGTTACCTTCGGCA | This work |
| qPCRgsu1963fw | CCCTGAGAGACCACGGTTTC | This work |
| qPCRgsu1963rv | CCCGAGAAAACCGACCAGTT | This work |
| qPCRgsu0470fw | GCGAGCCGTGATGATGACTA | This work |
| qPCRgsu0470rv | CAGGGGCTTGGTGAGGAAAT | This work |
| FwGSU1554 | TCGTCAGTTGAAGGATGTGG | This work |
| RevGSU1554 | TGAACATGATATCGGCATGG | This work |
| FwGSU2044 | ATTTCCGACGTGAAAGATGC | This work |
| RevGSU2044 | ATGCTCGTGGTGATGAACAA | This work |
| qPCRGSU0018fw | CTCCTACGATATGCCACCCG | This work |
| qPCRGSU0018rv | GAGGTGGTCACGATCTGGTC | This work |
| qPCRGSU3409fw | GGGTTCCTCGCTCCTCTTTT | This work |
| qPCRGSU3409rv | AAAAGCCCCTTCAGCATCGA | This work |
| qPCRGSU3410fw | TCTTGGGAGCGTAACATCGG | This work |
| qPCRGSU3410rv | ACGATGATCAGTGCGCAGAA | This work |
| qPCRGSU0597fw | CATTCGGCGCACTGATCATC | This work |
| qPCRGSU0597rv | CGATGTTCCGGTGGCGATAT | This work |

**References**

Andrade A, Hernández-Eligio A, Tirado AL, Vega-Alvarado L, Olvera M, Morett E, Juárez K. (2021). Specialization of the Reiterated Copies of the Heterodimeric Integration Host Factor Genes in *Geobacter sulfurreducens. Front Microbiol*. 12:626443.

Caccavo FJR, Lonergan DJ, Lovley DR, Davis M, Stolz JF, Mcinerney MJ. (1994). *Geobacter sulfurreducens* sp. nov., a Hydrogen- and Acetate- oxidizing dissimilatory metal-reducing microorganism. *Appl Environ Microbiol*. 3752-37589.

Hernández-Eligio A, Pat-Espadas AM, Vega-Alvarado L, Huerta-Amparán M, Cervantes FJ, Juárez K. (2020). Global transcriptional analysis of *Geobacter sulfurreducens* under palladium reducing conditions reveals new key cytochromes involved. *Appl Microbiol Biotech*. 104:4059-4069.

Holmes DE, Chaudhuri SK, Nevin KP, Mehta T, Methé BA, Liu A, Ward JE, Woodard TL, Webster J, Lovley DR. (2006). Microarray and genetic analysis of electron transfer to electrodes in *Geobacter sulfurreducens*. *Environ Microbiol*. 8:1805–1815.

Jaramillo-Rodriguez B, Vega-Alvarado L, Rodriguez-Torres LM, Huerta-Miranda G, Hernández-Eligio A, Juárez K. (2023). Global transcriptional analysis of *Geobacter sulfurreducens* *gsu1771* mutant biofilm grown on two different support structures. *PLoS ONE*. 18(10), e0293359.

Kim B-C, Leang C, Ding Y-H, Glaven R H, Coppi M V, Lovley DR. (2005). OmcF, a putative c-type monoheme outer membrane cytochrome required for the expression of other outer membrane cytochromes in *Geobacter sulfurreducens*. *J Bacteriol*. 187:4505–4513.

Simon R, Priefer U, Pühler A. (1983). A broad host range mobilization system for in vivo genetic engineering: transposon mutagenesis in gram negative bacteria. *Biotech*. 1:784–791.
