# Supplementary material for: The role of CsrA in controls the extracellular electron transfer and biofilm production in Geobacter sulfurreducens
Source: Front Microbiol. 2025 Mar 11;16:1534446. doi: 10.3389/fmicb.2025.1534446 (PMC11934962; doi:10.3389/fmicb.2025.1534446)
Supplement: Supplementary file 2 [file Table_2.docx]

**Supplementary Table 2** List of differentially expressed genes in Δ*csrA* compared with the DL1 strain during growth in glass.

| **Locus tag** | **Name** | **Expression** | **pValue** | **log2FC** |
| --- | --- | --- | --- | --- |
| **Energy metabolism and electron transport** | | | |  |
| GSU0614 | | Upregulated | 2.80E-06 | 1.9230218 |
| GSU0784 | hybB | Upregulated | 5.49E-14 | 1.7447879 |
| GSU0785 | hybL | Upregulated | 4.32E-15 | 1.984804 |
| GSU0786 | hybP | Upregulated | 1.37E-10 | 1.6349965 |
| GSU1640 | cydA | Upregulated | 1.35E-10 | 2.0323335 |
| GSU2748 | | Upregulated | 3.36E-28 | 3.3208047 |
| GSU2801 | | Upregulated | 2.39E-08 | 1.7794183 |
| GSU3274 | | Upregulated | 2.81E-23 | 4.7145039 |
| GSU0012 | hemY | Downregulated | 3.91E-18 | -2.492888 |
| GSU0067 | can-1 | Downregulated | 8.73E-21 | -2.229457 |
| GSU0068 | | Downregulated | 4.76E-12 | -1.940456 |
| GSU0364 | ppcB | Downregulated | 9.99E-47 | -5.241811 |
| GSU0466 | macA | Downregulated | 7.66E-14 | -1.742705 |
| GSU0593 | | Downregulated | 3.18E-20 | -2.271119 |
| GSU0594 | | Downregulated | 1.19E-14 | -1.994389 |
| GSU1877 | | Downregulated | 4.97E-50 | -3.574127 |
| GSU2811 | | Downregulated | 1.15E-11 | -1.565357 |
| GSU2883 | omcH | Downregulated | 0.0001132 | -1.586589 |
| GSU2908 | | Downregulated | 6.14E-07 | -2.195521 |
| GSU2912 | omcO | Downregulated | 1.13E-09 | -2.39201 |
| GSU2913 | omcP | Downregulated | 0.0001187 | -1.785622 |
| **Transport** | |  |  |  |
| GSU0321 | gspL | Upregulated | 8.49E-05 | 1.6994176 |
| GSU0325 | gspH | Upregulated | 0.0001163 | 2.1925453 |
| GSU0814 | | Upregulated | 7.00E-07 | 1.6164007 |
| GSU0972 | | Upregulated | 7.33E-34 | 2.7199471 |
| GSU1016 | | Upregulated | 0.0009409 | 1.7147576 |
| GSU1153 | | Upregulated | 4.89E-07 | 2.384792 |
| GSU1380 | feoB-1 | Upregulated | 1.30E-33 | 3.1785612 |
| GSU2135 | | Upregulated | 0.0071813 | 3.4890189 |
| GSU2340 | mrpE | Upregulated | 0.000747 | 1.8562636 |
| GSU2782 | | Upregulated | 8.87E-05 | 1.7024127 |
| GSU3070 | ftsW | Upregulated | 3.19E-08 | 1.6461157 |
| GSU3268 | feoB-2 | Upregulated | 4.11E-55 | 3.9228668 |
| GSU3269 | | Upregulated | 3.17E-28 | 3.7246663 |
| GSU3270 | | Upregulated | 7.71E-17 | 4.4370925 |
| GSU3271 | | Upregulated | 1.87E-41 | 3.7984519 |
| GSU3392 | | Upregulated | 8.87E-05 | 2.6738388 |
| GSU1557 | | Downregulated | 9.92E-13 | -1.747614 |
| GSU2005 | | Downregulated | 2.30E-28 | -2.523403 |
| GSU2697 | acrA | Downregulated | 1.31E-07 | -2.408088 |
| GSU3304 | ompJ | Downregulated | 6.98E-14 | -1.679405 |
| **Regulatory functions and transcription** | | | |  |
| GSU0470 | | Upregulated | 2.49E-07 | 2.4400471 |
| GSU0471 | | Upregulated | 4.02E-05 | 2.6614061 |
| GSU0776 | | Upregulated | 0.0008703 | 1.5662071 |
| GSU1379 | fur | Upregulated | 1.28E-18 | 2.5690897 |
| GSU1382 | ideR | Upregulated | 1.18E-18 | 2.7911127 |
| GSU2749 | | Upregulated | 4.54E-19 | 2.7001869 |
| GSU3263 | | Upregulated | 0.0084461 | 3.4124286 |
| GSU0013 | | Downregulated | 6.47E-12 | -2.500034 |
| GSU0018 | | Downregulated | 1.12E-13 | -2.144915 |
| GSU0266 | | Downregulated | 0.0001382 | -1.593507 |
| GSU0401 | mcp40H-12 | Downregulated | 7.82E-05 | -2.082437 |
| GSU0446 | rsmE | Downregulated | 1.31E-53 | -3.72356 |
| GSU0596 | | Downregulated | 2.08E-13 | -1.977346 |
| GSU0721 | rpoE | Downregulated | 6.65E-09 | -1.582167 |
| GSU1090 | | Downregulated | 1.55E-10 | -1.619438 |
| GSU1303 | mcp34H-11 | Downregulated | 7.97E-09 | -1.722921 |
| GSU1671 | | Downregulated | 1.60E-11 | -1.843037 |
| GSU2016 | | Downregulated | 2.51E-07 | -1.798108 |
| GSU2641 | | Downregulated | 7.88E-25 | -2.330901 |
| GSU2698 | | Downregulated | 4.91E-07 | -2.395063 |
| GSU2809 | | Downregulated | 5.46E-06 | -1.609675 |
| GSU2822 | gnfR | Downregulated | 3.12E-08 | -3.648402 |
| GSUR053 | | Downregulated | 0.0001124 | -1.641534 |
| GSU1727 | | Downregulated | 8.42E-11 | -1.650185 |
| GSU1746 | ihfB-1 | Downregulated | 4.34E-05 | -1.768291 |
| **DNA/RNA metabolism** | | |  |  |
| GSU0567 | tag | Upregulated | 0.0023127 | 3.4302726 |
| GSU1421 | sbcD-1 | Upregulated | 0.0014809 | 2.45567 |
| GSU2862 | rpoC | Upregulated | 7.68E-09 | 1.532508 |
| GSU2863 | rpoB | Upregulated | 1.04E-07 | 1.6008613 |
| GSU3266 | | Upregulated | 7.45E-24 | 2.6523302 |
| GSU0043 | | Downregulated | 0.0011415 | -2.004018 |
| GSU0546 | | Downregulated | 3.68E-05 | -1.756221 |
| GSU0547 | mutS-2 | Downregulated | 1.02E-37 | -3.170172 |
| GSU2614 | recJ | Downregulated | 7.60E-18 | -2.579515 |
| **Metabolism of cofactors and vitamins** | | | |  |
| GSU0686 | dxs-1 | Upregulated | 0.0002697 | 1.7934684 |
| GSU0380 | lipA | Upregulated | 1.19E-05 | 2.0732275 |
| GSU0215 | folD-1 | Downregulated | 3.27E-13 | -2.024183 |
| GSU1577 | cobA | Downregulated | 1.73E-10 | -2.121344 |
| **Unknown function** | |  |  |  |
| GSU0973 | | Upregulated | 2.87E-18 | 2.5722298 |
| GSU0974 | | Upregulated | 3.95E-26 | 2.668532 |
| GSU0977 | | Upregulated | 1.73E-28 | 3.0988948 |
| GSU0978 | | Upregulated | 1.19E-13 | 2.276239 |
| GSU0979 | | Upregulated | 3.77E-21 | 2.3899986 |
| GSU0980 | | Upregulated | 1.50E-31 | 2.708648 |
| GSU0981 | | Upregulated | 1.24E-21 | 2.2329553 |
| GSU0982 | | Upregulated | 4.98E-32 | 2.6420106 |
| GSU0983 | | Upregulated | 4.97E-21 | 2.5168066 |
| GSU0987 | | Upregulated | 1.25E-51 | 3.8376925 |
| GSU0988 | | Upregulated | 1.43E-38 | 3.102229 |
| GSU0992 | | Upregulated | 3.35E-38 | 3.6818315 |
| GSU1011 | | Upregulated | 0.0001225 | 2.4392789 |
| GSU0990 | | Upregulated | 5.83E-28 | 3.5786876 |
| GSU1253 | | Upregulated | 0.0009235 | 2.2142248 |
| GSU1846 | | Upregulated | 0.0001047 | 3.6195944 |
| GSU1969 | | Upregulated | 3.56E-06 | 2.5294462 |
| GSU1971 | | Upregulated | 2.55E-05 | 1.874975 |
| GSU2276 | | Upregulated | 0.0005921 | 1.5907614 |
| GSU2750 | | Upregulated | 1.19E-10 | 1.5499335 |
| GSU2968 | | Upregulated | 7.23E-13 | 2.2959948 |
| GSU3082 | | Upregulated | 5.68E-09 | 1.511374 |
| GSU3141 | | Upregulated | 4.02E-30 | 3.2781789 |
| GSU3267 | | Upregulated | 1.50E-25 | 3.9181558 |
| GS_RS16380 | | Upregulated | 6.33E-35 | 5.4738167 |
| GS_RS16385 | | Upregulated | 4.76E-33 | 4.1487553 |
| GS_RS17460 | | Upregulated | 1.41E-10 | 1.920978 |
| GSU2938 | | Downregulated | 1.42E-18 | -1.909801 |
| GSU0071 | | Downregulated | 5.79E-44 | -3.871136 |
| GSU0081 | | Downregulated | 2.31E-17 | -2.017521 |
| GSU0186 | | Downregulated | 1.90E-19 | -2.121506 |
| GSU0216 | | Downregulated | 3.60E-29 | -2.851861 |
| GSU0444 | | Downregulated | 2.23E-32 | -3.413386 |
| GSU0539 | | Downregulated | 1.00E-11 | -2.055562 |
| GSU0597 | | Downregulated | 8.04E-07 | -1.7486 |
| GSU0722 | | Downregulated | 1.39E-09 | -1.574589 |
| GSU0919 | | Downregulated | 5.05E-15 | -1.698397 |
| GSU1001 | | Downregulated | 6.02E-20 | -4.831547 |
| GSU1071 | | Downregulated | 4.20E-05 | -1.853044 |
| GSU1167 | | Downregulated | 1.12E-14 | -1.780565 |
| GSU1395 | | Downregulated | 2.22E-17 | -2.002306 |
| GSU1447 | | Downregulated | 6.12E-12 | -2.3406 |
| GSU1472 | | Downregulated | 5.16E-07 | -1.765761 |
| GSU1615 | | Downregulated | 2.53E-08 | -1.896106 |
| GSU1669 | | Downregulated | 5.00E-20 | -2.579758 |
| GSU1726 | | Downregulated | 1.43E-14 | -2.543267 |
| GSU1995 | | Downregulated | 2.53E-09 | -1.755303 |
| GSU2077 | | Downregulated | 1.05E-08 | -1.624114 |
| GSU2404 | | Downregulated | 5.27E-11 | -2.219973 |
| GSU2585 | | Downregulated | 1.27E-26 | -2.801077 |
| GSU2586 | | Downregulated | 1.43E-08 | -1.669691 |
| GSU2662 | | Downregulated | 1.41E-12 | -3.721808 |
| GSU2663 | | Downregulated | 6.13E-30 | -4.704758 |
| GSU2668 | | Downregulated | 2.75E-06 | -1.671877 |
| GSU2895 | | Downregulated | 0.0049768 | -1.621573 |
| GSU2902 | | Downregulated | 2.39E-09 | -3.286501 |
| GSU2907 | | Downregulated | 2.68E-09 | -2.367876 |
| GSU2911 | | Downregulated | 4.18E-05 | -1.727572 |
| GSU3351 | | Downregulated | 4.32E-30 | -2.360991 |
| GSU3409 | | Downregulated | 6.67E-39 | -2.851677 |
| GSU3410 | | Downregulated | 1.35E-32 | -3.133039 |
| GSU3425 | | Downregulated | 2.45E-09 | -1.938252 |
| GSU3452 | slyX | Downregulated | 6.47E-11 | -2.461742 |
| GSU3478 | | Downregulated | 1.08E-08 | -1.913452 |
| GSU3489 | | Downregulated | 1.83E-08 | -1.633443 |
| GSU3506 | | Downregulated | 4.04E-18 | -3.303979 |
| GSU3511 | | Downregulated | 7.35E-10 | -2.126596 |
| GSU3549 | | Downregulated | 3.82E-13 | -1.900708 |
| GSU3629 | | Downregulated | 0.0002329 | -3.25047 |
| **Others** |  |  |  |  |
| GSU0975 | | Upregulated | 3.22E-23 | 2.504279 |
| GSU0976 | | Upregulated | 2.16E-20 | 2.4601451 |
| GSU0985 | | Upregulated | 4.12E-17 | 2.9393377 |
| GSU0986 | | Upregulated | 1.58E-19 | 3.0305668 |
| GSU1154 | | Upregulated | 3.74E-14 | 1.8212192 |
| GSU1844 | | Upregulated | 0.0008396 | 2.6076242 |
| GSU1896 | kdsB | Upregulated | 0.0010194 | 1.5681244 |
| GSU1974 | | Upregulated | 3.74E-09 | 2.5377961 |
| GSU2519 | yjiM | Upregulated | 0.0011094 | 1.6491152 |
| GSU2967 | | Upregulated | 3.11E-08 | 1.5715414 |
| GSU3065 | ftsQ | Upregulated | 2.16E-10 | 1.8450908 |
| GSU3265 | | Upregulated | 0.0079119 | 2.8318483 |
| GSU0066 | | Downregulated | 1.80E-28 | -2.42835 |
| GSU0069 | | Downregulated | 2.12E-12 | -1.806912 |
| GSU0182 | | Downregulated | 2.17E-16 | -1.957014 |
| GSU0200 | | Downregulated | 3.01E-17 | -1.922413 |
| GSU0201 | | Downregulated | 1.72E-13 | -1.674882 |
| GSU0202 | | Downregulated | 9.96E-12 | -1.562316 |
| GSU0217 | | Downregulated | 5.82E-14 | -1.60308 |
| GSU0352 | prx-3 | Downregulated | 7.57E-28 | -2.534554 |
| GSU0472 | | Downregulated | 8.70E-09 | -1.582231 |
| GSU0490 | ato-1 | Downregulated | 4.82E-39 | -2.901783 |
| GSU0510 | sfrB | Downregulated | 9.04E-14 | -1.772915 |
| GSU0548 | | Downregulated | 1.42E-38 | -3.351503 |
| GSU0672 | | Downregulated | 6.75E-12 | -1.570139 |
| GSU0794 | | Downregulated | 5.29E-05 | -1.611616 |
| GSU0795 | | Downregulated | 0.0003124 | -2.178013 |
| GSU0884 | | Downregulated | 1.81E-06 | -2.261595 |
| GSU1168 | | Downregulated | 8.48E-07 | -1.633367 |
| GSU1471 | | Downregulated | 1.76E-22 | -2.631249 |
| GSU1496 | pilA-N | Downregulated | 6.35E-22 | -3.059253 |
| GSU1497 | pilA-C | Downregulated | 4.79E-26 | -3.30938 |
| GSU1614 | | Downregulated | 4.27E-11 | -1.812721 |
| GSU1670 | | Downregulated | 2.39E-18 | -2.270137 |
| GSU1711 | | Downregulated | 3.70E-24 | -2.704993 |
| GSU2885 | | Downregulated | 9.93E-08 | -1.739805 |
| GSU2896 | | Downregulated | 1.02E-05 | -1.705091 |
| GSU2905 | | Downregulated | 0.0009547 | -1.845091 |
| GSU2914 | | Downregulated | 1.74E-09 | -1.732612 |
| GSU2945 | | Downregulated | 0.0009358 | -1.981846 |
| GSU3014 | | Downregulated | 6.89E-14 | -4.680064 |
| GSU3040 | fliW | Downregulated | 2.54E-11 | -5.403692 |
| GSU3042 | flgL | Downregulated | 4.29E-22 | -5.781596 |
| GSU3043 | flgK | Downregulated | 3.82E-08 | -2.127901 |
| GSU3342 | | Downregulated | 1.09E-10 | -1.571436 |
| GSU3424 | lpdA-3 | Downregulated | 3.35E-09 | -1.630774 |
| GSU3451 | | Downregulated | 6.47E-26 | -2.47808 |
| **Amino acids metabolism** | | |  |  |
| GSU0989 | | Upregulated | 2.51E-38 | 3.3938459 |
| GSU3066 | ddl | Upregulated | 1.45E-11 | 1.7871038 |
| GSU3068 | murC | Upregulated | 3.98E-12 | 2.0029474 |
| GSU3142 | aroG-2 | Upregulated | 1.13E-31 | 3.5708564 |
| GSU1799 | | Upregulated | 0.000697 | 1.7617722 |
| GSU0936 | | Downregulated | 0.0033657 | -1.635435 |
| GSU1490 | aroE | Downregulated | 0.0003061 | -1.613644 |
| GSU2487 | cpkA | Downregulated | 3.20E-09 | -1.580096 |
| GSU3098 | hisB | Downregulated | 2.12E-05 | -1.57576 |
| GSU3395 | putA | Downregulated | 1.19E-10 | -1.965637 |
| **Cell envelope** | |  |  |  |
| GSU0991 | | Upregulated | 2.75E-34 | 3.5703565 |
| GSU1262 | | Upregulated | 0.0002411 | 3.1281833 |
| GSU1963 | | Upregulated | 0.0003041 | 2.480071 |
| GSU3072 | mraY | Upregulated | 3.03E-08 | 1.9020915 |
| GSU1473 | | Downregulated | 9.52E-05 | -1.711058 |
| GSU1551 | | Downregulated | 1.15E-12 | -3.321352 |
| GSU1943 | | Downregulated | 2.26E-10 | -3.369841 |
| **Nucleotide metabolism** | | |  |  |
| GSU1272 | pyrC | Upregulated | 0.007512 | 3.1398688 |
| GSU2236 | relA | Upregulated | 2.89E-05 | 2.5592935 |
| **Protein Synthesis** | |  |  |  |
| GSU1516 | infC | Upregulated | 0.000504 | 1.547879 |
| GSU3235 | rpmA | Downregulated | 3.47E-14 | -1.795822 |
| GSU3236 | rplU | Downregulated | 1.44E-12 | -1.743944 |
| GSUR013 | | Downregulated | 1.18E-11 | -1.834786 |
| GSUR020 | | Downregulated | 1.18E-11 | -1.835292 |
| **Lipid metabolism** | |  |  |  |
| GSU1603 | fabG-2 | Upregulated | 0.0067127 | 2.7817646 |
| GSU2584 | | Downregulated | 1.95E-26 | -2.462137 |
| **Carbohydrate metabolism** | | |  |  |
| GSU1962 | | Upregulated | 0.0034362 | 3.3433825 |
| GSU1970 | neuB | Upregulated | 0.0009395 | 1.7923188 |
| GSU1972 | | Upregulated | 8.72E-09 | 2.9298565 |
| GSU1973 | | Upregulated | 4.46E-05 | 2.1392684 |
| GSU1975 | | Upregulated | 5.16E-07 | 3.0342874 |
| GSU1976 | | Upregulated | 2.41E-12 | 2.8414891 |
| GSU1977 | | Upregulated | 4.02E-05 | 1.6254912 |
| GSU1978 | epsI | Upregulated | 0.0002948 | 3.4830872 |
| GSU1980 | | Upregulated | 1.41E-07 | 3.0118854 |
| GSU2366 | rmlB | Upregulated | 0.0035475 | 4.1859911 |
| GSU2428 | pyc | Upregulated | 1.54E-06 | 2.3549624 |
| GSU3069 | murG | Upregulated | 3.38E-10 | 1.725091 |
| GSU2918 | tklB | Upregulated | 0.0011512 | 1.6831246 |
| GSU1176 | frdC | Downregulated | 8.19E-21 | -2.44555 |
| GSU1177 | frdA | Downregulated | 1.16E-19 | -2.07486 |
| GSU1178 | frdB | Downregulated | 1.62E-08 | -1.650676 |
| GSU2302 | | Downregulated | 2.99E-13 | -2.170237 |
| **Signal transduction** | | |  |  |
| GSU1037 | | Downregulated | 1.19E-10 | -2.033472 |
| GSU1299 | cheW34H-2 | Downregulated | 0.0001585 | -2.002671 |
| GSU1554 | | Downregulated | 1.86E-05 | -2.889936 |
| GSU2044 | | Downregulated | 4.34E-17 | -2.807831 |
| GSU2667 | | Downregulated | 1.15E-10 | -1.583851 |
| **Proteolysis** | |  |  |  |
| GSU1944 | | Downregulated | 7.71E-17 | -4.373987 |
