# Supplementary material for: The role of CsrA in controls the extracellular electron transfer and biofilm production in Geobacter sulfurreducens
Source: Front Microbiol. 2025 Mar 11;16:1534446. doi: 10.3389/fmicb.2025.1534446 (PMC11934962; doi:10.3389/fmicb.2025.1534446)
Supplement: Supplementary file 3 [file Table_3.docx]

**Supplementary Table 3** List of differentially expressed genes in Δ*csrA* compared with the DL1 strain during energy production on MFC.

| **Locus Tag** | **Name** | **Regulation** | **pValue** | **log2FC** |
| --- | --- | --- | --- | --- |
| **Energy metabolism and electron transport** | | | | |
| GSU0012 | hemY | Upregulation | 1.71E-10 | 1.553 |
| GSU0670 | omcX | Upregulation | 0.000355 | 2.225 |
| GSU0702 | | Upregulation | 0.002619 | 1.744 |
| GSU0782 | hybS | Upregulation | 1.12E-20 | 2.111 |
| GSU0783 | hybA | Upregulation | 1.12E-18 | 2.816 |
| GSU0784 | hybB | Upregulation | 6.29E-20 | 2.743 |
| GSU0785 | hybL | Upregulation | 6.84E-21 | 4.267 |
| GSU0786 | hybP | Upregulation | 8.84E-24 | 3.437 |
| GSU1442 | | Upregulation | 1.03E-24 | 4.526 |
| GSU1538 | | Upregulation | 2.50E-06 | 2.412 |
| GSU2096 | cooF | Upregulation | 1.23E-28 | 4.411 |
| GSU2098 | cooS | Upregulation | 1.31E-87 | 6.378 |
| GSU2294 | omcM | Upregulation | 5.47E-08 | 3.815 |
| GSU2513 | | Upregulation | 5.59E-12 | 2.426 |
| GSU2801 | | Upregulation | 5.00E-09 | 1.916 |
| GSU2808 | | Upregulation | 1.63E-09 | 2.038 |
| GSU1394 | ompB | Downregulation | 6.25E-24 | -2.63 |
| GSU1877 | | Downregulation | 6.98E-50 | -2.452 |
| GSU2503 | omcT | Downregulation | 8.54E-10 | -5.546 |
| GSU2504 | omcS | Downregulation | 5.53E-31 | -4.945 |
| GSU3214 | | Downregulation | 5.36E-07 | -3.158 |
| **DNA/RNA metabolism** | | |  |  |
| GSU0057 | cas1-1_cas4 | Upregulation | 0.003768 | 1.533 |
| GSU3279 | uvrC | Upregulation | 4.49E-05 | 1.522 |
| GSU0547 | mutS-2 | Downregulation | 4.95E-30 | -1.976 |
| GSU2614 | recJ | Downregulation | 1.44E-12 | -2.534 |
| **Transport** | |  |  |  |
| GSU0433 | tssH | Upregulation | 1.34E-06 | 2.813 |
| GSU0575 | cstA | Upregulation | 1.28E-05 | 1.751 |
| GSU0706 | | Upregulation | 0.006173 | 1.716 |
| GSU0707 | sugE | Upregulation | 0.001966 | 2.47 |
| GSU0972 | | Upregulation | 7.43E-63 | 2.977 |
| GSU1153 | | Upregulation | 3.29E-07 | 2.126 |
| GSU1279 | nikMN | Upregulation | 0.001107 | 2.043 |
| GSU1380 | feoB-1 | Upregulation | 2.54E-11 | 1.597 |
| GSU2781 | | Upregulation | 0.000217 | 1.609 |
| GSU2782 | | Upregulation | 2.16E-06 | 1.775 |
| GSU2950 | | Upregulation | 1.36E-06 | 1.845 |
| GSU0212 | | Downregulation | 0.001606 | -1.696 |
| GSU0828 | | Downregulation | 8.20E-08 | -2.82 |
| GSU0829 | | Downregulation | 1.73E-07 | -3.187 |
| GSU0830 | | Downregulation | 0.001558 | -3.26 |
| GSU2135 | | Downregulation | 1.80E-13 | -3.054 |
| GSU3304 | ompJ | Downregulation | 4.85E-17 | -1.518 |
| GSU3404 | | Downregulation | 0.003684 | -1.544 |
| **Unknown function** | |  |  |  |
| GSU0516 | | Upregulation | 2.45E-06 | 1.721 |
| GSU0603 | | Upregulation | 0.004094 | 2.081 |
| GSU0714 | | Upregulation | 7.27E-21 | 1.65 |
| GSU0715 | | Upregulation | 5.91E-15 | 1.582 |
| GSU0788 | | Upregulation | 4.16E-11 | 2.071 |
| GSU0973 | | Upregulation | 1.85E-28 | 2.456 |
| GSU0974 | | Upregulation | 5.46E-56 | 2.971 |
| GSU0977 | | Upregulation | 2.20E-40 | 2.716 |
| GSU0978 | | Upregulation | 2.23E-13 | 2.013 |
| GSU0980 | | Upregulation | 1.35E-25 | 1.796 |
| GSU0981 | | Upregulation | 7.19E-60 | 2.613 |
| GSU0982 | | Upregulation | 5.74E-31 | 1.954 |
| GSU0983 | | Upregulation | 7.64E-13 | 1.591 |
| GSU0987 | | Upregulation | 1.23E-58 | 2.793 |
| GSU0988 | | Upregulation | 9.45E-24 | 2.353 |
| GSU0990 | | Upregulation | 4.22E-58 | 3.514 |
| GSU0992 | | Upregulation | 1.24E-35 | 2.968 |
| GSU1081 | | Upregulation | 8.40E-05 | 1.568 |
| GSU1082 | | Upregulation | 3.67E-11 | 2.206 |
| GSU1269 | | Upregulation | 3.64E-06 | 4.842 |
| GSU2295 | | Upregulation | 0.000422 | 2.158 |
| GSU2478 | | Upregulation | 7.91E-15 | 5.138 |
| GSU2561 | | Upregulation | 8.50E-06 | 1.813 |
| GSU2968 | | Upregulation | 1.60E-60 | 5.177 |
| GSU3141 | | Upregulation | 2.02E-15 | 2.615 |
| GSU3414 | | Upregulation | 0.002365 | 2.782 |
| GSU3509 | | Upregulation | 0.004054 | 1.66 |
| GSU0071 | | Downregulation | 3.97E-62 | -2.973 |
| GSU0081 | | Downregulation | 2.35E-07 | -1.587 |
| GSU0216 | | Downregulation | 3.14E-18 | -1.898 |
| GSU0444 | | Downregulation | 0.000201 | -1.67 |
| GSU0919 | | Downregulation | 1.11E-32 | -1.892 |
| GSU1339 | | Downregulation | 1.33E-07 | -1.594 |
| GSU1395 | | Downregulation | 7.12E-24 | -3.254 |
| GSU1500 | | Downregulation | 2.12E-05 | -2.615 |
| GSU1512 | | Downregulation | 5.50E-06 | -2.153 |
| GSU1620 | | Downregulation | 1.19E-08 | -1.584 |
| GSU1669 | | Downregulation | 3.87E-11 | -1.525 |
| GSU1948 | | Downregulation | 2.56E-11 | -1.62 |
| GSU2143 | | Downregulation | 1.06E-05 | -2.933 |
| GSU2505 | | Downregulation | 2.95E-18 | -5.019 |
| GSU2585 | | Downregulation | 4.13E-12 | -1.637 |
| GSU2640 | | Downregulation | 6.27E-11 | -2.37 |
| GSU2662 | | Downregulation | 2.51E-09 | -4.356 |
| GSU2663 | | Downregulation | 3.71E-80 | -4.99 |
| GSU2938 | | Downregulation | 0.000463 | -1.712 |
| GSU3084 | | Downregulation | 1.77E-12 | -1.804 |
| GSU3568 | lnt-C | Downregulation | 3.64E-08 | -3.145 |
| GSU3629 | | Downregulation | 1.14E-15 | -2.447 |
| **Proteolysis** | |  |  |  |
| GSU1944 | | Upregulation | 5.80E-17 | 1.712 |
| GSU2717 | hoxP | Upregulation | 0.002976 | 1.857 |
| GSU0896 | tldD | Downregulation | 7.19E-60 | -3.151 |
| **Regulatory functions and transcription** | | | |  |
| GSU0013 | | Upregulation | 0.000151 | 2.012 |
| GSU0470 | | Upregulation | 0.000417 | 2.849 |
| GSU0471 | | Upregulation | 1.91E-07 | 2.733 |
| GSU0475 | | Upregulation | 6.76E-13 | 1.773 |
| GSU1148 | | Upregulation | 3.42E-05 | 1.794 |
| GSU1264 | | Upregulation | 5.20E-05 | 7.05 |
| GSU1265 | | Upregulation | 3.28E-09 | 3.351 |
| GSU1999 | hfq | Upregulation | 4.74E-14 | 1.954 |
| GSU2480 | kdpA | Upregulation | 1.73E-09 | 1.789 |
| GSU2670 | | Upregulation | 0.001757 | 2.692 |
| GSU3261 | | Upregulation | 1.87E-30 | 2.711 |
| GSU3419 | | Upregulation | 0.002243 | 2.331 |
| GSUR056 | | Upregulation | 0.000295 | 1.709 |
| GSU1746 | ihfB-1 | Downregulation | 7.06E-05 | -1.693 |
| GSU2214 | cheB40H | Downregulation | 9.85E-05 | -1.655 |
| GSU2442 | | Downregulation | 1.52E-05 | -2.625 |
| GSU2506 | | Downregulation | 9.99E-12 | -2.982 |
| GSU2507 | | Downregulation | 5.93E-07 | -2.31 |
| GSU2641 | | Downregulation | 7.82E-27 | -2.464 |
| GSU2815 | | Downregulation | 2.28E-07 | -2.081 |
| **Others** |  |  |  |  |
| GSU0136 | | Upregulation | 0.001233 | 2.09 |
| GSU0182 | | Upregulation | 2.37E-09 | 1.639 |
| GSU0515 | usp-1 | Upregulation | 7.76E-05 | 1.598 |
| GSU0544 | | Upregulation | 2.98E-23 | 1.893 |
| GSU0819 | | Upregulation | 3.26E-05 | 2.069 |
| GSU0975 | | Upregulation | 1.81E-49 | 2.545 |
| GSU0976 | | Upregulation | 1.66E-30 | 2.233 |
| GSU0985 | | Upregulation | 7.47E-19 | 2.339 |
| GSU0986 | | Upregulation | 2.81E-27 | 2.487 |
| GSU1154 | | Upregulation | 1.73E-32 | 2.051 |
| GSU1556 | | Upregulation | 9.21E-12 | 2.307 |
| GSU2095 | | Upregulation | 2.03E-129 | 6.071 |
| GSU2097 | cooC | Upregulation | 3.92E-47 | 6.361 |
| GSU2560 | | Upregulation | 0.000102 | 1.631 |
| GSU2562 | sixA | Upregulation | 2.52E-07 | 2.163 |
| GSU2814 | | Upregulation | 9.58E-07 | 1.802 |
| GSU2967 | | Upregulation | 1.74E-18 | 4.532 |
| GSU3030 | | Upregulation | 8.34E-06 | 2.408 |
| GSU0448 | | Downregulation | 1.53E-05 | -1.93 |
| GSU0548 | | Downregulation | 3.44E-20 | -1.928 |
| GSU0884 | | Downregulation | 0.000113 | -1.911 |
| GSU0930 | | Downregulation | 0.000178 | -1.617 |
| GSU1235 | | Downregulation | 5.64E-09 | -2.296 |
| GSU1237 | | Downregulation | 0.001392 | -1.709 |
| GSU1496 | pilA-N | Downregulation | 6.13E-27 | -2.556 |
| GSU1497 | pilA-C | Downregulation | 4.57E-43 | -2.498 |
| GSU1498 | xapA | Downregulation | 3.80E-06 | -2.159 |
| GSU2034 | pilX-2 | Downregulation | 1.60E-06 | -2.236 |
| GSU2035 | pilW-2 | Downregulation | 1.52E-12 | -1.783 |
| GSU2036 | pilV-2 | Downregulation | 4.75E-09 | -1.991 |
| GSU2038 | pilY1-2 | Downregulation | 2.46E-18 | -1.707 |
| GSU2896 | | Downregulation | 1.51E-05 | -1.736 |
| GSU3085 | yqfO | Downregulation | 2.41E-11 | -1.626 |
| GSU3329 | | Downregulation | 8.42E-13 | -1.611 |
| GSU3542 | | Downregulation | 1.72E-05 | -2.156 |
| **Amino acids metabolism** | | |  |  |
| GSU0375 | gcvT | Upregulation | 0.000345 | 1.516 |
| GSU0376 | gcvH-1 | Upregulation | 0.000231 | 1.532 |
| GSU0989 | | Upregulation | 5.80E-64 | 3.281 |
| GSU3142 | aroG-2 | Upregulation | 1.44E-15 | 2.144 |
| GSU2487 | cpkA | Downregulation | 1.71E-10 | -1.919 |
| GSU3096 | hisA | Downregulation | 6.94E-05 | -1.678 |
| GSU3097 | hisH | Downregulation | 9.04E-07 | -1.874 |
| GSU3098 | hisB | Downregulation | 6.47E-10 | -2.053 |
| **Carbohydrate metabolism** | | |  |  |
| GSU0818 | | Upregulation | 0.000111 | 1.719 |
| GSU1962 | | Upregulation | 4.87E-07 | 2.472 |
| GSU1176 | frdC | Downregulation | 1.47E-13 | -1.739 |
| **Cell envelope** | |  |  |  |
| GSU0991 | | Upregulation | 3.82E-39 | 3.142 |
| GSU1943 | | Upregulation | 3.52E-15 | 1.504 |
| GSU1855 | | Downregulation | 2.51E-10 | -2.924 |
| GSU2039 | pilL | Downregulation | 1.11E-06 | -2.828 |
| **Metabolism of cofactors and vitamins** | | | |  |
| GSU2290 | | Upregulation | 1.41E-05 | 1.862 |
| GSU1184 | acpH | Downregulation | 0.000454 | -1.927 |
| **Lipid metabolism** | |  |  |  |
| GSU2329 | | Upregulation | 6.75E-15 | 2.447 |
| GSU3029 | | Upregulation | 1.20E-21 | 3.046 |
| **Signal transduction** | |  |  |  |
| GSU0895 | | Downregulation | 1.68E-14 | -2.401 |
| GSU1037 | | Downregulation | 2.57E-15 | -3.611 |
| GSU2044 | | Downregulation | 5.84E-08 | -1.849 |
| **Nucleotide metabolism** | | |  |  |
| GSU1717 | cysD | Downregulation | 0.000214 | -1.63 |
| GSU1718 | cysN | Downregulation | 1.70E-11 | -1.675 |
| **Protein synthesis** | |  |  |  |
| GSU1833 | trpS | Downregulation | 0.002136 | -1.577 |
| GSU2843 | rpsH | Downregulation | 7.08E-11 | -1.554 |
| GSU2844 | rpsN | Downregulation | 3.89E-09 | -1.605 |
| GSU2845 | rplE | Downregulation | 4.04E-13 | -1.532 |
| GSU2848 | rpsQ | Downregulation | 2.82E-10 | -1.668 |
| GSU2853 | rpsS | Downregulation | 3.76E-10 | -1.514 |
| GSU3611 | rpmJ | Downregulation | 6.32E-06 | -1.723 |
