# Supplementary material for: The role of CsrA in controls the extracellular electron transfer and biofilm production in Geobacter sulfurreducens
Source: Front Microbiol. 2025 Mar 11;16:1534446. doi: 10.3389/fmicb.2025.1534446 (PMC11934962; doi:10.3389/fmicb.2025.1534446)
Supplement: Supplementary file 4 [file Table_4.docx]

**Supplementary Table 4** List of differentially expressed genes only in graphite electrode biofilms of Δ*csrA* strain with respect to DL1.

| **Locus Tag** | **Name** | **Regulation** | **pValue** | **log2FC** |
| --- | --- | --- | --- | --- |
| **Energy metabolism and electron transport** | | | | |
| GSU0670 | omcX | Upregulation | 0.000355 | 2.225 |
| GSU0702 |  | Upregulation | 0.002619 | 1.744 |
| GSU0782 | hybS | Upregulation | 1.12E-20 | 2.111 |
| GSU0783 | hybA | Upregulation | 1.12E-18 | 2.816 |
| GSU1442 |  | Upregulation | 1.03E-24 | 4.526 |
| GSU1538 |  | Upregulation | 2.50E-06 | 2.412 |
| GSU2096 | cooF | Upregulation | 1.23E-28 | 4.411 |
| GSU2098 | cooS | Upregulation | 1.31E-87 | 6.378 |
| GSU2294 | omcM | Upregulation | 5.47E-08 | 3.815 |
| GSU2513 |  | Upregulation | 5.59E-12 | 2.426 |
| GSU2808 |  | Upregulation | 1.63E-09 | 2.038 |
| GSU1394 | ompB | Downregulation | 6.25E-24 | -2.63 |
| GSU2503 | omcT | Downregulation | 8.54E-10 | -5.546 |
| GSU2504 | omcS | Downregulation | 5.53E-31 | -4.945 |
| GSU3214 |  | Downregulation | 5.36E-07 | -3.158 |
| **DNA/RNA metabolism** | | | | |
| GSU0057 | cas1-1_cas4 | Upregulation | 0.003768 | 1.533 |
| GSU3279 | uvrC | Upregulation | 4.49E-05 | 1.522 |
| **Transport** | | | | |
| GSU0433 | tssH | Upregulation | 1.34E-06 | 2.813 |
| GSU0575 | cstA | Upregulation | 1.28E-05 | 1.751 |
| GSU0706 |  | Upregulation | 0.006173 | 1.716 |
| GSU0707 | sugE | Upregulation | 0.001966 | 2.47 |
| GSU1279 | nikMN | Upregulation | 0.001107 | 2.043 |
| GSU2781 |  | Upregulation | 0.000217 | 1.609 |
| GSU2950 |  | Upregulation | 1.36E-06 | 1.845 |
| GSU0212 |  | Downregulation | 0.001606 | -1.696 |
| GSU0828 |  | Downregulation | 8.20E-08 | -2.82 |
| GSU0829 |  | Downregulation | 1.73E-07 | -3.187 |
| GSU0830 |  | Downregulation | 0.001558 | -3.26 |
| GSU3404 |  | Downregulation | 0.003684 | -1.544 |
| **Unknown function** | | | | |
| GSU0516 |  | Upregulation | 2.45E-06 | 1.721 |
| GSU0603 |  | Upregulation | 0.004094 | 2.081 |
| GSU0714 |  | Upregulation | 7.27E-21 | 1.65 |
| GSU0715 |  | Upregulation | 5.91E-15 | 1.582 |
| GSU0788 |  | Upregulation | 4.16E-11 | 2.071 |
| GSU1081 |  | Upregulation | 8.40E-05 | 1.568 |
| GSU1082 |  | Upregulation | 3.67E-11 | 2.206 |
| GSU1269 |  | Upregulation | 3.64E-06 | 4.842 |
| GSU2295 |  | Upregulation | 0.000422 | 2.158 |
| GSU2478 |  | Upregulation | 7.91E-15 | 5.138 |
| GSU2561 |  | Upregulation | 8.50E-06 | 1.813 |
| GSU3414 |  | Upregulation | 0.002365 | 2.782 |
| GSU3509 |  | Upregulation | 0.004054 | 1.66 |
| GSU1339 |  | Downregulation | 1.33E-07 | -1.594 |
| GSU1500 |  | Downregulation | 2.12E-05 | -2.615 |
| GSU1512 |  | Downregulation | 5.50E-06 | -2.153 |
| GSU1620 |  | Downregulation | 1.19E-08 | -1.584 |
| GSU1948 |  | Downregulation | 2.56E-11 | -1.62 |
| GSU2143 |  | Downregulation | 1.06E-05 | -2.933 |
| GSU2505 |  | Downregulation | 2.95E-18 | -5.019 |
| GSU2640 |  | Downregulation | 6.27E-11 | -2.37 |
| GSU3084 |  | Downregulation | 1.77E-12 | -1.804 |
| GSU3568 | lnt-C | Downregulation | 3.64E-08 | -3.145 |
| **Proteolysis** | | | | |
| GSU2717 | hoxP | Upregulation | 0.002976 | 1.857 |
| GSU0896 | tldD | Downregulation | 7.19E-60 | -3.151 |
| **Regulatory functions and transcription** | | | | |
| GSU0475 |  | Upregulation | 6.76E-13 | 1.773 |
| GSU1148 |  | Upregulation | 3.42E-05 | 1.794 |
| GSU1264 |  | Upregulation | 5.20E-05 | 7.05 |
| GSU1265 |  | Upregulation | 3.28E-09 | 3.351 |
| GSU1999 | hfq | Upregulation | 4.74E-14 | 1.954 |
| GSU2480 | kdpA | Upregulation | 1.73E-09 | 1.789 |
| GSU2670 |  | Upregulation | 0.001757 | 2.692 |
| GSU3261 |  | Upregulation | 1.87E-30 | 2.711 |
| GSU3419 |  | Upregulation | 0.002243 | 2.331 |
| GSUR056 |  | Upregulation | 0.000295 | 1.709 |
| GSU2214 | cheB40H | Downregulation | 9.85E-05 | -1.655 |
| GSU2442 |  | Downregulation | 1.52E-05 | -2.625 |
| GSU2506 |  | Downregulation | 9.99E-12 | -2.982 |
| GSU2507 |  | Downregulation | 5.93E-07 | -2.31 |
| GSU2815 |  | Downregulation | 2.28E-07 | -2.081 |
| **Others** | | | | |
| GSU0136 |  | Upregulation | 0.001233 | 2.09 |
| GSU0515 | usp-1 | Upregulation | 7.76E-05 | 1.598 |
| GSU0544 |  | Upregulation | 2.98E-23 | 1.893 |
| GSU0819 |  | Upregulation | 3.26E-05 | 2.069 |
| GSU1556 |  | Upregulation | 9.21E-12 | 2.307 |
| GSU2095 |  | Upregulation | 2.03E-129 | 6.071 |
| GSU2097 | cooC | Upregulation | 3.92E-47 | 6.361 |
| GSU2560 |  | Upregulation | 0.000102 | 1.631 |
| GSU2562 | sixA | Upregulation | 2.52E-07 | 2.163 |
| GSU2814 |  | Upregulation | 9.58E-07 | 1.802 |
| GSU3030 |  | Upregulation | 8.34E-06 | 2.408 |
| GSU0448 |  | Downregulation | 1.53E-05 | -1.93 |
| GSU0930 |  | Downregulation | 0.000178 | -1.617 |
| GSU1235 |  | Downregulation | 5.64E-09 | -2.296 |
| GSU1237 |  | Downregulation | 0.001392 | -1.709 |
| GSU1498 | xapA | Downregulation | 3.80E-06 | -2.159 |
| GSU2034 | pilX-2 | Downregulation | 1.60E-06 | -2.236 |
| GSU2035 | pilW-2 | Downregulation | 1.52E-12 | -1.783 |
| GSU2036 | pilV-2 | Downregulation | 4.75E-09 | -1.991 |
| GSU2038 | pilY1-2 | Downregulation | 2.46E-18 | -1.707 |
| GSU3085 | yqfO | Downregulation | 2.41E-11 | -1.626 |
| GSU3329 |  | Downregulation | 8.42E-13 | -1.611 |
| GSU3542 |  | Downregulation | 1.72E-05 | -2.156 |
| **Amino acids metabolism** | | | | |
| GSU0375 | gcvT | Upregulation | 0.000345 | 1.516 |
| GSU0376 | gcvH-1 | Upregulation | 0.000231 | 1.532 |
| GSU3096 | hisA | Downregulation | 6.94E-05 | -1.678 |
| GSU3097 | hisH | Downregulation | 9.04E-07 | -1.874 |
| **Carbohydrate metabolism** | | | | |
| GSU0818 |  | Upregulation | 0.000111 | 1.719 |
| **Cell envelope** | | | | |
| GSU1855 |  | Downregulation | 2.51E-10 | -2.924 |
| GSU2039 | pilL | Downregulation | 1.11E-06 | -2.828 |
| **Metabolism of cofactors and vitamins** | | | | |
| GSU2290 |  | Upregulation | 1.41E-05 | 1.862 |
| GSU1184 | acpH | Downregulation | 0.000454 | -1.927 |
| **Lipid metabolism** | | | | |
| GSU2329 |  | Upregulation | 6.75E-15 | 2.447 |
| GSU3029 |  | Upregulation | 1.20E-21 | 3.046 |
| **Signal transduction** | | | | |
| GSU0895 |  | Downregulation | 1.68E-14 | -2.401 |
| **Nucleotide metabolism** | | | | |
| GSU1717 | cysD | Downregulation | 0.000214 | -1.63 |
| GSU1718 | cysN | Downregulation | 1.70E-11 | -1.675 |
| **Protein synthesis** | | | | |
| GSU1833 | trpS | Downregulation | 0.002136 | -1.577 |
| GSU2843 | rpsH | Downregulation | 7.08E-11 | -1.554 |
| GSU2844 | rpsN | Downregulation | 3.89E-09 | -1.605 |
| GSU2845 | rplE | Downregulation | 4.04E-13 | -1.532 |
| GSU2848 | rpsQ | Downregulation | 2.82E-10 | -1.668 |
| GSU2853 | rpsS | Downregulation | 3.76E-10 | -1.514 |
| GSU3611 | rpmJ | Downregulation | 6.32E-06 | -1.723 |
