# Supplementary material for: The role of CsrA in controls the extracellular electron transfer and biofilm production in Geobacter sulfurreducens
Source: Front Microbiol. 2025 Mar 11;16:1534446. doi: 10.3389/fmicb.2025.1534446 (PMC11934962; doi:10.3389/fmicb.2025.1534446)
Supplement: Supplementary file 5 [file Table_5.docx]

**Supplementary Table 5** Genes with putative CsrA binding site located in the 5´-untranslated region with expression changes in RNA-seq analysis.

|  |  |  |  | **Regulation in Δ*csrA* strain** | |
| --- | --- | --- | --- | --- | --- |
| Locus Tag | Description | Position* | Sequence | MFC | Glass |
| GSU0012 | *hemG,* protoporphyrinogen oxidase | -5 | GGAAGGAGAG | Upregulated | Downregulated |
| GSU0186 | Hipotetical protein | -40 | GAAAGGAGAU | ND | Downregulated |
| GSU0200 | *iorA,* isoquinoline 1-oxidoreductase | -5 | AAAAGGAGGC | ND | Downregulated |
| GSU0216 | Hipotetical protein | -5 | GAAAGGAGUA | Downregulated | Downregulated |
| GSU0217 | Nitroreductase family protein | -6 | ACAAGGAGGC | ND | Downregulated |
| GSU0352 | *psaD,* thiol peroxidase | -5 | ACAAGGAGGA | ND | Downregulated |
| GSU0364 | *cyd-1,* cytochrome c3 | -5 | GAAAGGAGAA | ND | Downregulated |
| GSU0376 | *gcvH-1,* glycine cleavage system H protein | -7 | ACAAGGAGGC | Upregulated | ND |
| GSU0444 | Hipotetical protein | -6 | ACAAGGAGUU | Downregulated | Downregulated |
| GSU0548 | Radical SAM domain protein | -60 | AGAAGGAGUA | Downregulated | Downregulated |
| GSU0594 | *cbcA,* cytochrome c family protein | -5 | UCAAGGAGGC | ND | Downregulated |
| GSU0603 | Hipotetical protein | -43 | GAAAGGAGAU | Upregulated | ND |
| GSU0670 | *omcX,* cytochrome c family protein | -3 | CAAAGGAGGA | Upregulated | ND |
| GSU0672 | Hipotetical protein | -4 | GAAAGGAGGA | ND | Downregulated |
| GSU0714 | Hipotetical protein | -7 | GAAAGGAGAG | Upregulated | Downregulated |
| GSU0721 | *rpoE,* RNA polymerase sigma -70 factor | -1 | GCAAGGAGAA | ND | Downregulated |
| GSU0786 | *hybP,* hydrogenase maturation protease | -65 | GCAAGGAGAU | Upregulated | Upregulated |
| GSU0828 | Metal ion efflux membrane protein | -27 | GCAAGGAGGU | Downregulated | ND |
| GSU0919 | Hipotetical protein | -2 | GGAAGGAGGC | Downregulated | Downregulated |
| GSU0930 | Sulfur transferase, putative | -11 | UCAAGGAGGC | Downregulated | ND |
| GSU0975 | Phage tail sheath protein, putative | -7 | GAAAGGAGGA | Upregulated | Upregulated |
| GSU0985 | Hipotetical protein | -4 | UGAAGGAGAU | Upregulated | Upregulated |
| GSU0990 | Hipotetical protein | -4 | AAAAGGAGGA | Upregulated | Upregulated |
| GSU1001 | Hipotetical protein | -5 | GGAAGGAGAC | ND | Downregulated |
| GSU1071 | Hipotetical protein | -7 | GAAAGGAGUA | ND | Downregulated |
| GSU1082 | Hipotetical protein | -78 | CCAAGGAGAU | Upregulated | ND |
| GSU1167 | Hipotetical protein | -8 | GAAAGGAGGG | ND | Downregulated |
| GSU1237 | Pyridine nucleotide-disulphide oxidoreductase family protein | -9 | ACAAGGAGGG | Downregulated | ND |
| GSU1269 | Hipotetical protein | -3 | ACAAGGAGGC | Upregulated | ND |
| GSU1394 | *ompB,* spore coat protein-related protein | -8 | CAAAGGAGAC | Downregulated | ND |
| GSU1395 | Hipotetical protein | -6 | AAAAGGAGAA | Downregulated | Downregulated |
| GSU1472 | Hipotetical protein | -4 | CAAAGGAGAU | ND | Downregulated |
| GSU1496 | *pilA-N,* pilin domain protein | -1 | GAAAGGAGAC | Downregulated | Downregulated |
| GSU1538 | Methylamine utilization protein MauG, putative | -97 | GAAAGGAGGG | Upregulated | Upregulated |
| GSU1556 | Hipotetical protein | -4 | GAAAGGAGUG | Upregulated | ND |
| GSU1557 | Mechanosensitive ion channel family protein | -5 | UCAAGGAGGA | ND | Downregulated |
| GSU1620 | Iron-sulfur cluster binding protein, putative | -6 | CGAAGGAGAA | Downregulated | ND |
| GSU1640 | *cydA,* cytochrome d ubiquinol oxidase, subunit I | -8 | CAAAGGAGGU | ND | Upregulated |
| GSU1670 | Lipoprotein, putative | -8 | CAAAGGAGGU | ND | Downregulated |
| GSU1799 | Aspartate kinase | -7 | CAAAGGAGGA | ND | Upregulated |
| GSU1844 | IPT/TIG domain protein, putative | -7 | GAAAGGAGAU | ND | Upregulated |
| GSU1855 | Capsule polysaccharide export protein, putative | -134 | ACAAGGAGUU | Downregulated | ND |
| GSU1943 | Hipotetical protein | -4 | ACAAGGAGAU | Upregulated | Downregulated |
| GSU1962 | Glycosyl transferase, group 2 family protein | -107 | UGAAGGAGGG | Upregulated | Upregulated |
| GSU1970 | *neuB,* polysaccharide biosynthesis protein, putative | -3 | AAAAGGAGAC | ND | Upregulated |
| GSU2005 | Branched-chain amino acid ABC transporter | -5 | CCAAGGAGGC | ND | Downregulated |
| GSU2035 | *pilW-2* | -135 | CAAAGGAGUA | Downregulated | ND |
| GSU2077 | Hipotetical protein | -3 | CAAAGGAGAA | ND | Downregulated |
| GSU2143 | Hipotetical protein | -5 | CAAAGGAGAA | Downregulated | ND |
| GSU2404 | Pentapeptide repeat domain protein | -5 | CCAAGGAGGC | ND | Downregulated |
| GSU2561 | Hipotetical protein | -4 | GGAAGGAGAG | Upregulated | ND |
| GSU2585 | Hipotetical protein | -4 | AAAAGGAGAU | Downregulated | Downregulated |
| GSU2586 | Hipotetical protein | -7 | AGAAGGAGGU | ND | Downregulated |
| GSU2663 | Lipoprotein, putative | -7 | GAAAGGAGGC | Downregulated | Downregulated |
| GSU2801 | Cytochrome c family protein | -4 | UCAAGGAGGA | Upregulated | Upregulated |
| GSU2811 | Hsc, cytochrome c Hsc | -4 | ACAAGGAGGA | ND | Downregulated |
| GSU2814 | Rubrerythrin | -6 | GAAAGGAGAG | Upregulated | ND |
| GSU2863 | *rpoB,* DNA-directed RNA polymerase, beta subunit | -3 | CGAAGGAGAA | ND | Upregulated |
| GSU2968 | Hipotetical protein | -4 | GAAAGGAGGA | Upregulated | Upregulated |
| GSU3030 | Hipotetical protein | -31 | CAAAGGAGGA | Upregulated | |
| GSU3040 | *fliW* | -5 | GGAAGGAGUA | ND | Downregulated |
| GSU3043 | *flgK,* flagellar hook-associated protein FlgK | -5 | GGAAGGAGAU | ND | Downregulated |
| GSU3065 | *ftsQ,* cell division protein FtsQ | -93 | UCAAGGAGUC | ND | Upregulated |
| GSU3082 | ISGsu7, transposase OrfA | -5 | UCAAGGAGGU | ND | Upregulated |
| GSU3263 | Response regulator | -115 | GCAAGGAGAU | ND | Upregulated |
| GSU3265 | Sulfite reductase, assimilatory-type | -3 | AAAAGGAGUA | ND | Upregulated |
| GSU3270 | *feoA,* family protein | -3 | GAAAGGAGUU | ND | Upregulated |
| GSU3271 | Hipotetical protein | -3 | ACAAGGAGGG | ND | Upregulated |
| GSU3304 | *ompJ,* LamB porin family protein, putative | -4 | UGAAGGAGGC | Downregulated | Downregulated |
| GSU3329 | Radical SAM domain protein | -132 | UCAAGGAGGG | Downregulated | ND |
| GSU3351 | Hipotetical protein | -7 | GAAAGGAGAU | ND | Downregulated |
| GSU3395 | *putA,* proline dehydrogenase/delta-1-pyrroline-5-carboxylate dehydrogenase | -4 | CGAAGGAGUG | ND | Downregulated |
| GSU3410 | Hipotetical protein | -4 | GAAAGGAGAC | ND | Downregulated |
| GSU3414 | Hipotetical protein | -99 | GGAAGGAGAG | Upregulated | ND |
| GSU3489 | Hipotetical protein | -7 | CGAAGGAGGA | ND | Downregulated |
| GSU3511 | Hipotetical protein | -7 | GAAAGGAGUC | ND | Downregulated |

*With respect to the first translation codon

MFC is Microbial Fuel Cells
