# Supplementary material for: The role of CsrA in controls the extracellular electron transfer and biofilm production in Geobacter sulfurreducens
Source: Front Microbiol. 2025 Mar 11;16:1534446. doi: 10.3389/fmicb.2025.1534446 (PMC11934962; doi:10.3389/fmicb.2025.1534446)
Supplement: Supplementary file 6 [file Data_Sheet_1.docx]

**SUPPLEMENTARY FIGURES**

**The role of CsrA in controls the extracellular electron transfer and biofilm production in *Geobacter sulfurreducens***

**Alberto Hernández-Eligio,^1,4^**^*^ **Leticia Vega-Alvarado,^2^ Xinying Liu,^3^ Jessica Cholula-Calixto,^1^ Guillermo Huerta-Miranda,^1^ Juárez Katy^1^**^#^

^1^Departamento de Microbiología Molecular, Instituto de Biotecnología, Universidad Nacional Autónoma de México, Cuernavaca, Morelos, México.

^2^Instituto de Ciencias Aplicadas y Tecnología, Universidad Nacional Autónoma de México, Ciudad Universitaria, Ciudad de México, México.

^3^Beijing Key Laboratory for Source Control Technology of Water Pollution, Engineering Research Center for Water Pollution Source Control and Eco-remediation, College of Environmental Science and Engineering, Beijing Forestry University, Beijing 100083, China.

^4^Investigador por México, CONAHCYT.

**Correspondence:**

*[alberto.hernandez@ibt.unam.mx](mailto:alberto.hernandez@ibt.unam.mx)

#[katy.juarez@ibt.unam.mx](mailto:katy.juarez@ibt.unam.mx)

**
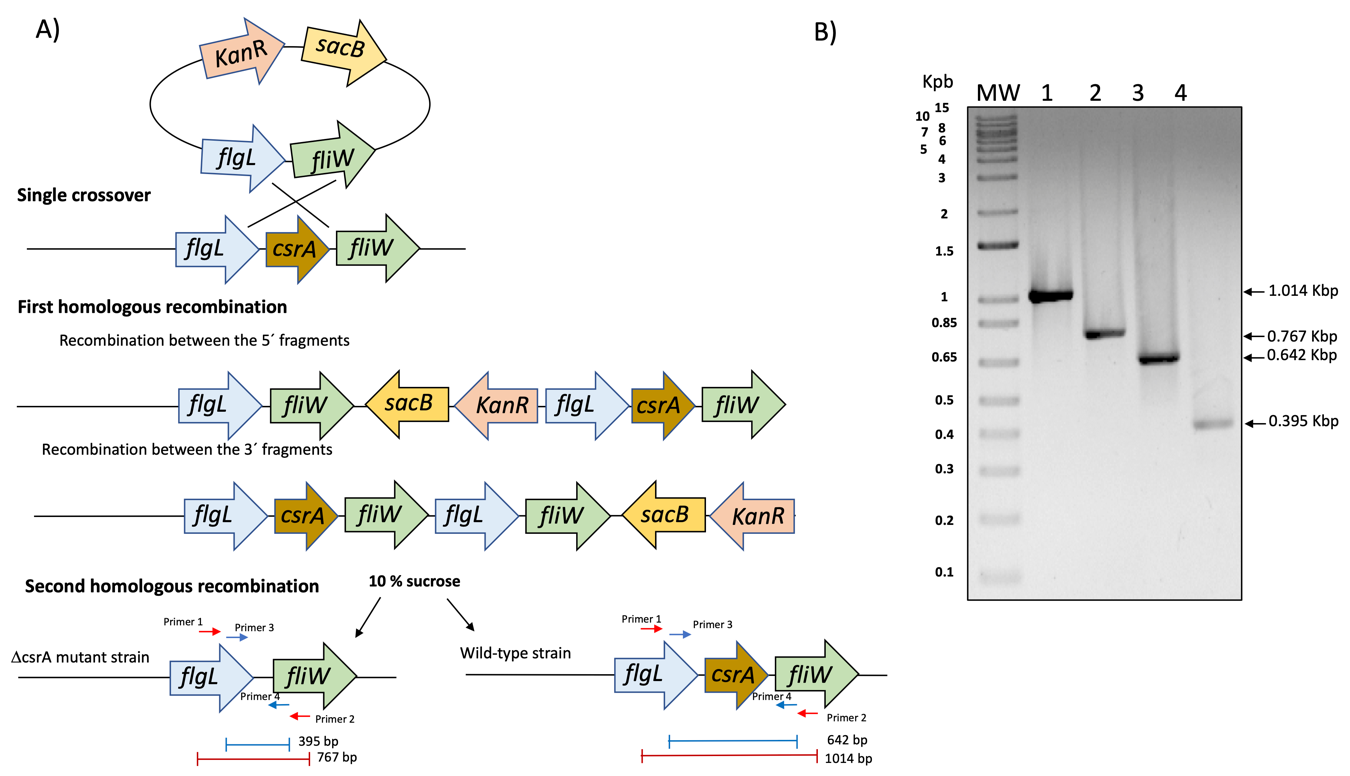
**

**Supplementary Figure 1.** A) Diagram of construction of Δ*csrA* strain of *G. sulfurreducens*. B) Gel with PCR fragments of *csrA* locus. Arrows showed the primers used and expected sizes. Lines 1 and 2 PCRs result using DNA of wild type and Δ*csrA* strains using 1, 2 primers, respectively. Lines 3 and 4, PCRs results using DNA of wild type and Δ*csrA* strains using 3, 4 primers, respectively. 1 Kb Plus DNA ladder was used as molecular weight (Invitrogen). Arrows indicate expected molecular weights.


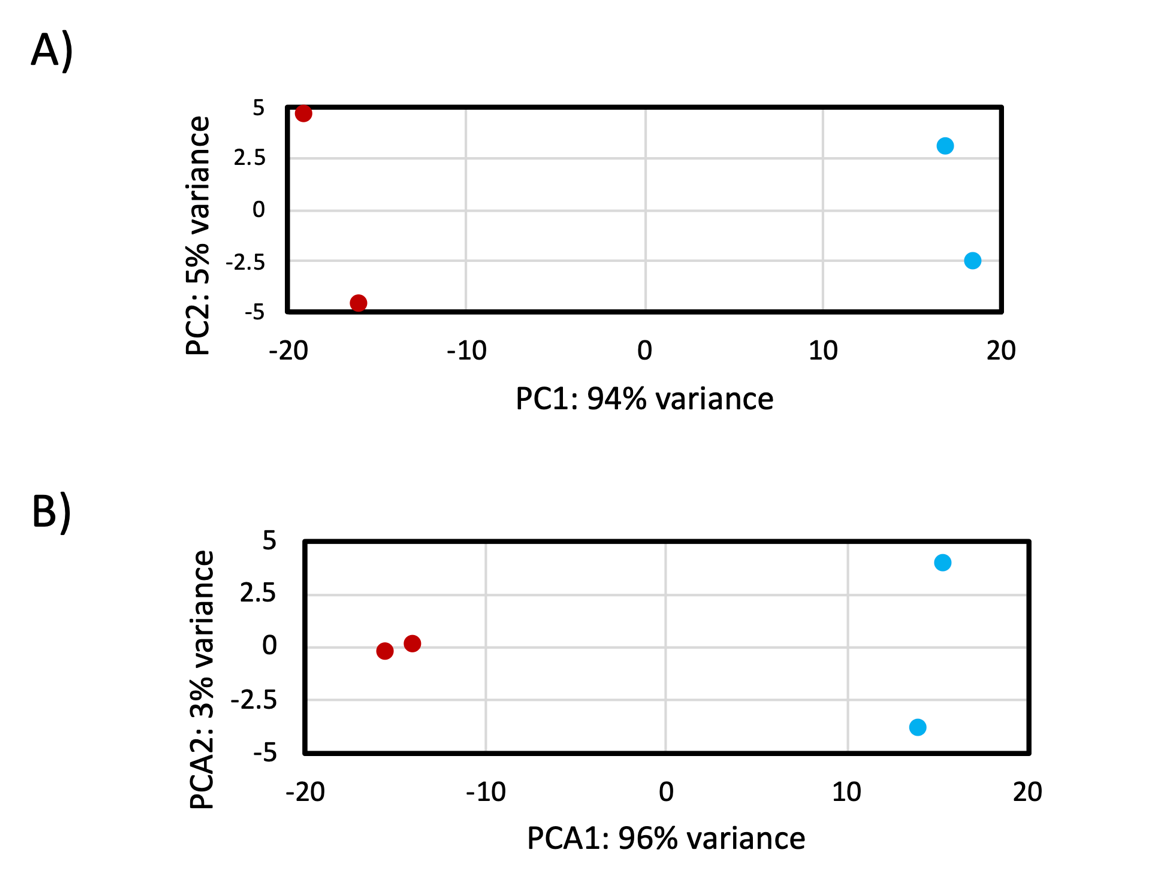


**Supplementary Figure 2.** Principal component analysis. A) Glass. B) Electrode. Δ*csrA* red circle and DL1 blue circle.


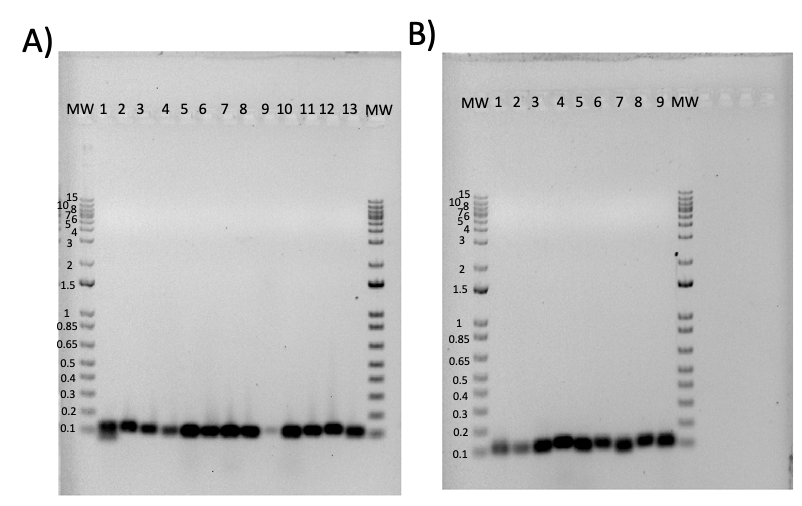


**Supplementary Figure 3.** Amplification of selected genes for RT-qPCR analysis. A) Lines 1-13 show the specific amplification of GSU1970 (*neuB*), GSU0470, GSU0597, GSU2236 (*relA*), GSU1944, GSU3014, GSU3410, GSU3268, GSU2822, GSU2044, GSU0364 (*ppcB*), GSU1554, and GSU0018 respectively. B) Lines 1-9 show the specific amplification of GSU3409, GSU3247, GSU0972, GSU2883 (*omcH*), GSU1496 (*pilA*), GSU3142 (*aroG-2*), GSU0490, GSU2236 and GSU1963 respectively. In A) and B) The molecular weight (MW) used is 1 kb DNA Ladder Plus (Thermo).


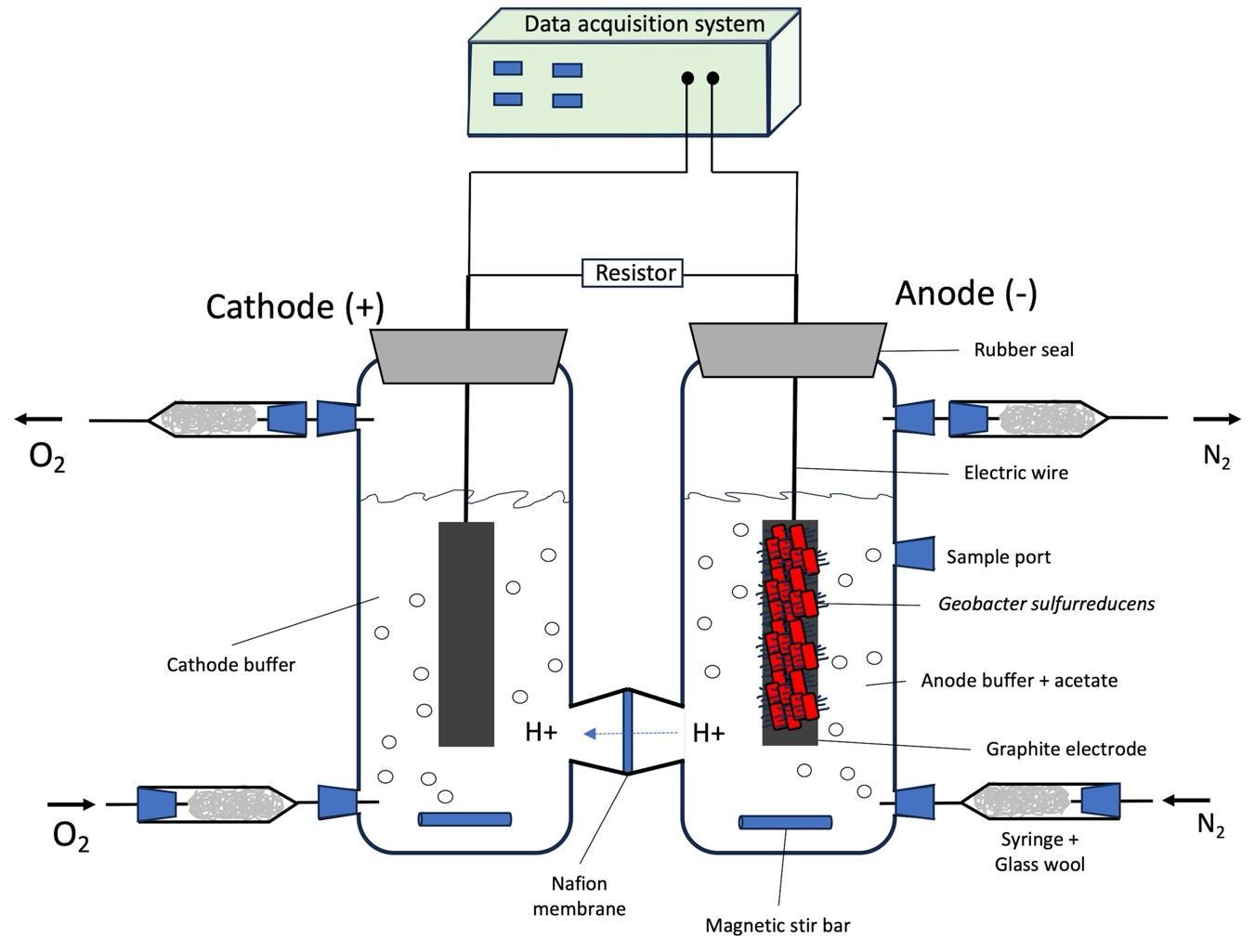


**Supplementary Figure 4.** Schematic diagram of the MFC used in this work.


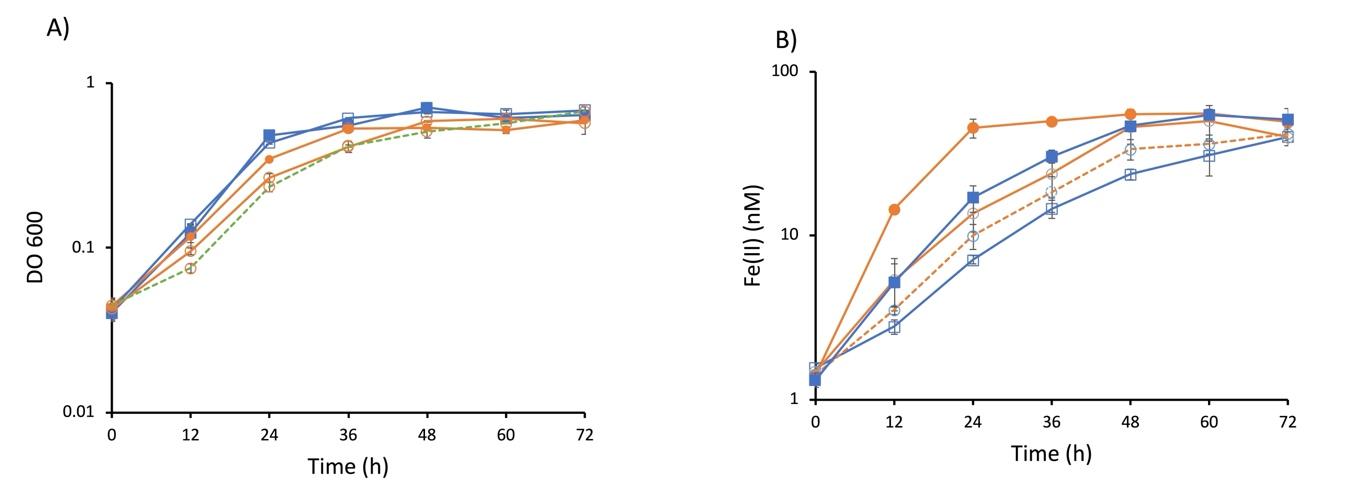


**Supplementary Figure 5.** Complementation of the Δ*csrA* mutant strain. A) Growth on acetate and fumarate (donor and electron acceptor, respectively). B) Soluble Fe(III) reduction in *csrA* complementation. DL1 blue line with filled squares, DL1/pGR5.1 blue line with empty squares, Δ*csrA* orange line with filled circle, Δ*csrA*/pRG5.1 orange line with empty circle, and DcsrA/pRG5.1-RRflg-*csrA* dashed orange line with empty circle.


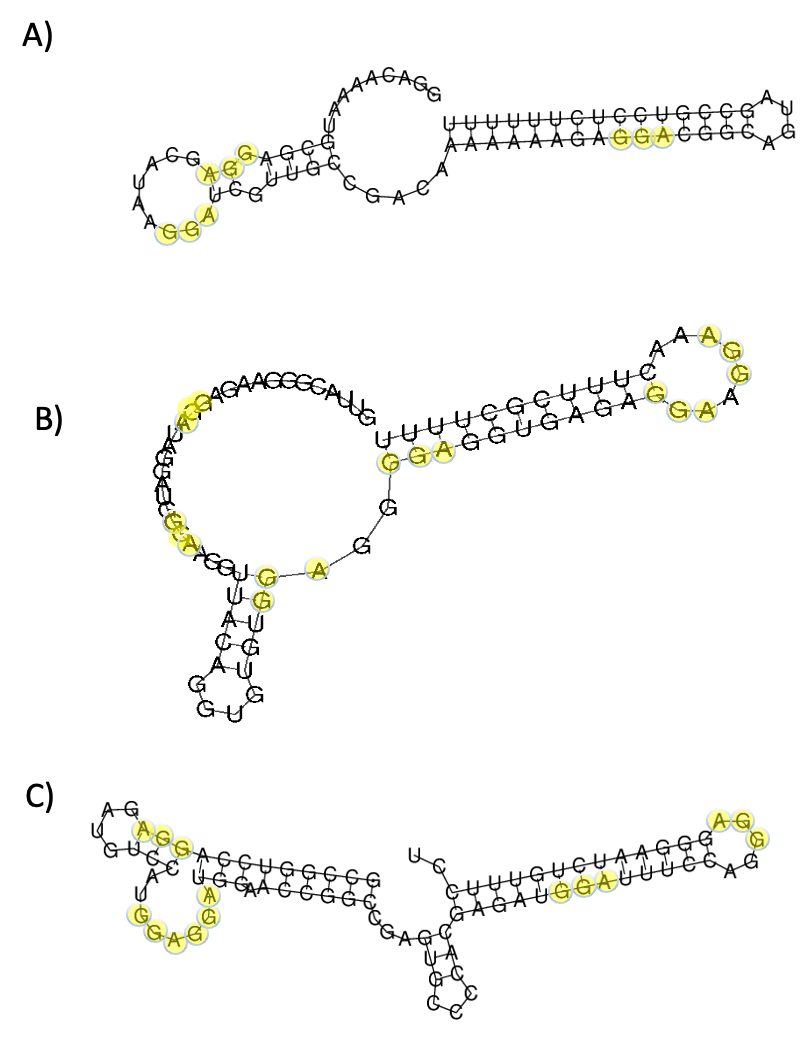


**Supplementary Figure 6.** Predicted sRNAs of the CsrB/CsrC family in *Geobacter sulfurreducens* PCA strain. A) Predicted secondary structure of the first sRNA (sRNA-1, 71 ribonucleotides) encoded in the antisense of the GSU0072 gene. The gene is located at coordinates 87387-87457 in the genome. B) Predicted secondary structure of the second sRNA (sRNA-2, 75 ribonucleotides) encoded between the GSU1100 and *phoR* genes. The gene is located at positions 1185706-1185780 in the genome. C) Predicted secondary structure of the third sRNA (sRNA-3, 82 ribonucleotides) encoded in the antisense of the GSU2675 gene. The gene is located at positions 2953737-2953818 in the genome. In each panel, the GGA motif that may be part of the possible CsrA binding site is highlighted with yellow circles.


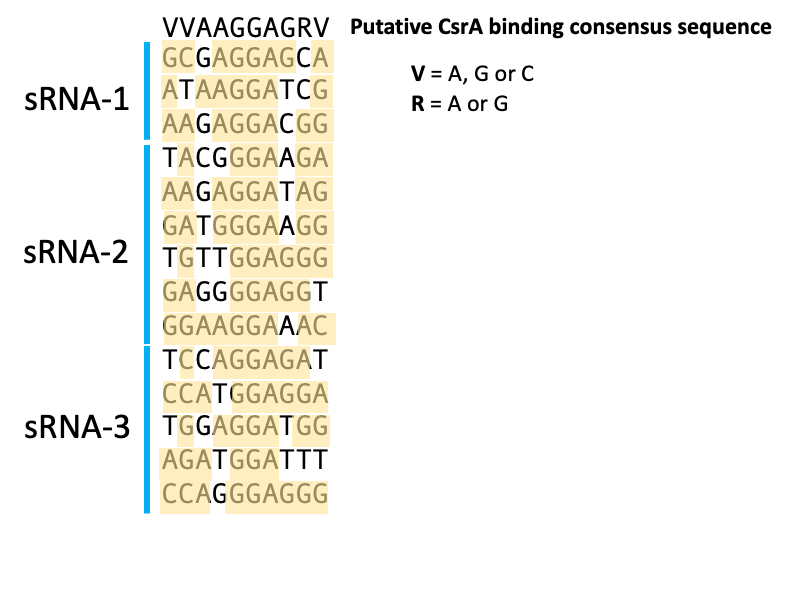


**Supplementary Figure 7.** Sequence analysis of the predicted sRNAs of the *Gebacter sulfurreducens* CsrB/CsrC family. The putative consensus CsrA binding sequence identified by MEME is shown at the top. The putative CsrA binding sites in the predicted sRNAs are shown. Nucleotides highlighted in yellow show the conserved bases relative to the consensus identified by MEME. In the consensus generated by MEME, V is A, G or C and R is A or G.
